# Down-regulation of respiration in pear fruit depends on temperature

**DOI:** 10.1093/jxb/ery031

**Published:** 2018-01-31

**Authors:** Quang Tri Ho, Maarten L A T M Hertog, Pieter Verboven, Alemayehu Ambaw, Seppe Rogge, Bert E Verlinden, Bart M Nicolaï

**Affiliations:** 1 KU Leuven, BIOSYST-MeBioS, Willem de Croylaan, Leuven, Belgium; 2 Flanders Centre of Postharvest Technology, Willem de Croylaan, Leuven, Belgium

**Keywords:** Diffusion, down, regulation, hypoxia, modelling, *Pyrus communis*, respiration

## Abstract

The respiration rate of plant tissues decreases when the amount of available O_2_ is reduced. There is, however, a debate on whether the respiration rate is controlled either by diffusion limitation of oxygen or through regulatory processes at the level of the transcriptome. We used experimental and modelling approaches to demonstrate that both diffusion limitation and metabolic regulation affect the response of respiration of bulky plant organs such as fruit to reduced O_2_ levels in the surrounding atmosphere. Diffusion limitation greatly affects fruit respiration at high temperature, but at low temperature respiration is reduced through a regulatory process, presumably a response to a signal generated by a plant oxygen sensor. The response of respiration to O_2_ is time dependent and is highly sensitive, particularly at low O_2_ levels in the surrounding atmosphere. Down-regulation of the respiration at low temperatures may save internal O_2_ and relieve hypoxic conditions in the fruit.

## Teaser

The respiration rate inside plant organs such as pear fruit is controlled both by diffusion limitation of oxygen and through additional regulatory processes in a temperature-dependent manner.

## Introduction

Gas exchange in plant organs relies on diffusion, causing gas to move from a high to a low concentration according to Fick’s law. Limited gas diffusion inside bulky organs (roots, fruits and tubers) may affect metabolic processes such as respiration, and lead to metabolic changes (Denison, 1992; Drew, 1997; Geigenberger *et al.*, 2000; Franck *et al.*, 2007; Ho *et al.*, 2010*a*; Armstrong and Beckett, 2011*a*; Verboven *et al.*, 2012). In seeds, restriction of gas diffusion during development or germination may result in hypoxic conditions (Rolletschek *et al.*, 2003; Borisjuk and Rolletschek, 2009; Verboven *et al.*, 2013) locally affecting respiratory activity. In fruit, the high resistance to gas transport of cortex tissue and the high respiration rate associated with ripening induces local anoxia during controlled atmosphere (CA) storage (Lammertyn *et al.*, 2003; Ho *et al.*, 2010*a*, 2013). In such anoxic stress conditions, metabolism is likely to switch from the respiratory to the fermentation pathway, causing physiological disorders (Franck *et al.*, 2007; Herremans *et al.*, 2013; Ho *et al.*, 2013).

Decreasing respiratory O_2_ consumption in response to a reduction in the available O_2_ has been described in different plant tissues, including seeds (van Dongen *et al.*, 2004), fruit (Lammertyn *et al.*, 2001, 2003; Ho *et al.*, 2010*b*, 2011), and roots (Geigenberger *et al.*, 2000; Gupta *et al.*, 2009; Zabalza *et al.*, 2009; Armstrong and Beckett, 2011*a*). While the respiratory metabolism is likely to be affected by local anoxia due to limited gas transport (Armstrong and Beckett, 2011*a*), active regulation of the respiratory metabolism may also play a role (Gupta *et al.*, 2009; Zabalza *et al.*, 2009). The arguments for both hypotheses have been reviewed (Gupta *et al.*, 2009; Armstrong and Beckett, 2011*b*; Nikoloski and van Dongen, 2011). Changes in the respiratory metabolism might also be explained by a combination of both mechanisms (Gupta *et al.*, 2009); however, this has not yet been investigated in detail.

As invasive measurement of gas concentrations in pear fruit is difficult without distorting and damaging the tissue, and thus affecting the gas exchange process itself, mathematical models provide a useful alternative. Such reaction–diffusion-type models have been commonly constructed by combining Michaelis–Menten respiration kinetics with Fick’s diffusion equation (Lammertyn *et al.*, 2003; Ho *et al.*, 2008, 2011, 2016; Armstrong and Beckett, 2011*a*). In earlier modelling works, diffusion coefficients and respiration parameters were explicitly and experimentally measured (Lammertyn *et al.*, 2003; Ho *et al.*, 2008). Armstrong and Beckett (2011*a*) did not experimentally determine diffusion coefficients and respiration rate, but estimated these from the data by means of the model describing O_2_ diffusion of roots using a multicylindrical geometry. As the experimental measurement of diffusion coefficients is prone to errors due to artefacts induced by cutting of the samples (flooding the intercellular spaces by leaking of the cell content), Ho *et al.* (2011, 2016) developed a method to compute these coefficients using microscale diffusion models and 3-D synchrotron X-ray tomography images of the tissue. For the respiration kinetics, the maximal rates were considered to vary with temperature while the Michaelis–Menten constants were shown to be relatively independent of temperature (Hertog *et al.*, 1998). Lammertyn *et al.*, (2001) estimated a $K_{m,O_{2}}$ value of 3 μM (0.14 kPa O_2_) for pear cell protoplasts. Armstrong and Beckett (2011*a*) used a $K_{m,O_{2}}$ value of 0.14 μM (0.011 kPa O_2_), averaged from values cited for isolated mitochondria from several plant species. Ho *et al.* (2013) estimated the $K_{m,O_{2}}$ of apple tissue to be within the range 0.13–0.17 kPa. The maximal rate coefficients in the Michaelis–Menten expression are typically obtained by fitting the model to respiration measurements (Hertog *et al.*, 1998; Lammertyn *et al.*, 2001; Armstrong and Beckett, 2011*a*; Ho *et al.*, 2013). Simulations with these reaction–diffusion models consistently predicted the existence of considerable gas gradients in the fruit, and local O_2_ concentrations in the centre of the fruit approaching the *K*_m_ of cytochrome *c* oxidase (COX), the rate-limiting enzyme in the oxidative respiration pathway. This suggests a passive regulation of the respiratory metabolism, but without excluding other regulatory mechanisms, for example at the transcriptome level as a consequence of a putative oxygen sensor. Such an oxygen sensor has been found recently in *Arabidopsis thaliana* (Licausi *et al.*, 2011), but its existence has yet to be shown in pear fruit. Typically, diffusion limitation is more pronounced at higher temperatures when the respiration rate is high. If the temperature is reduced (i.e. from 20 °C to 0 °C), the respiratory activity decreases correspondingly (Ho *et al.*, 2013). Any gradients of the O_2_ concentration in the fruit would then be reduced and the O_2_ concentration would become close to uniform throughout the fruit. This provides direct experimental control over internal O_2_ concentration of the uniform fruit by changing the fruit external atmosphere, thereby potentially exposing other regulatory mechanisms of the respiration pathway.

In this article, we aim to improve our understanding of the response of the oxidative respiratory metabolism of pear fruit to external O_2_ levels by challenging our previously developed gas exchange model (Ho *et al.*, 2010*a*, 2011) with new experimental data on pear respiration. The objectives, therefore, were (i) to evaluate whether changes of the respiratory activity under hypoxic conditions are due to diffusion limitation and/or active down-regulation; and (ii) to update our modelling concepts regarding the response of respiration to O_2_ concentrations at low temperature accordingly. We consider pear as it has a dense tissue with a high diffusion resistance inducing large internal gas gradients. Also, pears are commercially stored under low oxygen conditions that aggravate hypoxic conditions inside the fruit.

## Materials and methods

### Fruit

Pear fruit (*Pyrus communis* L. cv. ‘Conference’) were picked from the experimental orchard of the Research Station of pcfruit (Velm, Belgium) on 9 September 2010 and 24 August 2014. In 2016 independent validations, fruit which were harvested on 12 September 2016 were purchased commercially from a local fruit co-operative. For the experiments in 2010, fruit were cooled and stored under ambient air at –1 °C. For the experiments in 2014, fruit were cooled and stored according to commercial protocols for a period of 21 d at –1 °C followed by CA conditions (2.5 kPa O_2_, 0.7 kPa CO_2_, –1 °C). For the experiment in 2016, fruit were stored for 2 months under regular air at –1 °C. Picking data and cooling procedures were according to optimal commercial practices used for long-term storage of fruit determined by the Flanders Centre of Postharvest Technology (VCBT, Belgium).

### Respiration measurements

A first experiment (Experiment A) was used to determine the maximal O_2_ consumption rate and the maximal fermentative CO_2_ production rate of fruit at different temperatures (see Supplementary Table S1 at *JXB* online). Fruit were placed in 1.7 litre glass jars (two fruit per jar ~0.43 litres) and flushed for 24 h. The gas mixture contained 21 kPa O_2_, 0 kPa CO_2_, and 79 kPa N_2_ for measuring the maximal O_2_ consumption rate, and 0 kPa O_2_, 0 kPa CO_2_, and 100 kPa N_2_ for measuring the maximal fermentative CO_2_ production rate. The experiment was carried out at 20, 10, and 0 °C. Four repetitions were carried out following the methodology of Ho *et al.* (2010*b*). In brief, flushing was arrested after 24 h, jars were closed, and changes of the O_2_ and CO_2_ partial pressures were measured over time by a gas analyser (Checkmate II, PBI, Dansensor, Denmark). The gas partial pressures were converted to molar concentrations following the ideal gas law. The respiration rate was calculated from the difference in gas concentration and the time lag between two measurements, and expressed in μmol per unit fruit volume (m^3^) per time (s).

Experiments B and C were used to determine kinetic parameters relating the response of respiration to O_2_ level. In experiment B, we investigated the response of respiration to abruptly changing O_2_ levels at low temperature. Samples were taken from CA storage and stored under regular air at 0 °C for 1 d before starting the experiment. Fruit were placed in 1.7 litre glass jars (two fruit per jar) at 0 °C at O_2_ levels that were dynamically varied during a period of 18 d measuring the respiration rate daily. After each respiration measurement, the flushing was restarted. During the first 3 d, the O_2_ level was set to 20 kPa. Then the O_2_ level was reduced to 15 kPa O_2_ (experiment B1) and 5 kPa O_2_ (experiment B2), respectively. After 13 d, the O_2_ level was increased again to 20 kPa. The temperature and CO_2_ level were kept at 0 °C and 0 kPa, respectively. Three repetitions were carried out. In addition, an experiment (B3) was conducted to measure the respiration rate (two repetitions) under dynamically changing O_2_ levels from 20 kPa to 8, 4, 20, and 4 kPa after 2, 4, 6, and 14 d, respectively (Supplementary Table S1).

In experiment C, we investigated changes in respiration when the O_2_ was slowly decreasing. Samples were taken from fruit stored under CA conditions (2.5 kPa O_2_, 0.7 kPa CO_2_, –1°C). Fruit were placed in 1.7 litre glass jars (two fruit per jar) and flushed with a gas mixture of 7 kPa O_2_, 0 kPa CO_2_ at 0 °C for 24 h. Then, the jars were closed and the changing gas conditions were measured during 14 d from which the respiration rate was calculated. The air pressures in the closed jars were also monitored by a pressure sensor (DPI 142, GE Druck, Germany, accuracy ±0.01%). Three repetitions were carried out.

Experiment D was carried out to validate the model. Samples were taken from fruit stored under normal atmosphere conditions (21 kPa O_2_, 0 kPa CO_2_, –1°C). Fruit were placed in 1.7 litre glass jars (two fruit per jar) and flushed with a gas mixture for 24 h. The mixture contained 21 kPa O_2_, 0 kPa CO_2_, and 79 kPa N_2_ for the experiments at 20 °C (D3, D5) and 10 °C (D1, D4, D6), 11 kPa O_2_, 0 kPa CO_2_, and 89 kPa N_2_ for the experiments at 5 °C (D7), and 7 kPa O_2_, 0 kPa CO_2_, and 93 kPa N_2_ for the experiments at 0 °C (D2, D8). Then, the jars were closed and the changing gas conditions were measured during 6, 10, 14, and 15 d for the experiments at 20, 10, 5, and 0 °C, respectively. Four replicate measurements were carried out. We used available respiration data of pear harvested in 2010 (Ho *et al.*, 2015) and data of pear harvested in 2014 and 2016 for validations.

In all cases, fruit were stored in the dark inside cold rooms during the incubation period for the various respiration measurements.

### Reaction–diffusion model for intact fruit

A previously developed reaction–diffusion model (Ho *et al.*, 2008, 2011; Verboven *et al.*, 2012) was used to describe the overall gas exchange of intact fruit to the externally applied O_2_ level (Ho *et al.*, 2008, 2011; Verboven *et al.*, 2012):

$$\alpha_{i}\frac{\partial C_{i}}{\partial t}=\nabla \times D_{i}\nabla C_{i}+R_{i}$$ (1)

with α_*i*_ the gas capacity of component *i* (O_2_ and CO_2_) of the tissue (Ho *et al.*, 2008, 2011; Verboven *et al.*, 2012), *C*_*i*_ (µmol m^−3^) the concentration of component *i*, *D*_*i*_ (m^2^ s^−1^) the apparent diffusion coefficient, *R*_*i*_ (mol m^−3^ s^−1^) the production term of gas component *i* related to O_2_ consumption or CO_2_ production, ∇ (m^−1^) the gradient operator, and *t* (s) time. Based on preliminary calculations, we found that permeation could be neglected. The gas capacity α_*i*_ is defined as (Ho *et al.*, 2008, 2011; Verboven *et al.*, 2012):

$$\alpha_{i}=\varepsilon +\left(1-\varepsilon \right)\times R\times T\times H_{i}=\frac{C_{i,tissue}}{C_{i}}$$ (2)

where ε is the fractional porosity of tissue, and *C*_*i*_ (µmol m^−3^) and *C*_*i*,tissue_ (µmol m^−3^) are the concentration of gas component *i* in the gas phase and the tissue, respectively. The concentration of the compound in the liquid phase of fruit tissue normally follows Henry’s law represented by constant *H*_*i*_ (mol m^−3^ Pa^−1^). *R* (8.314 J mol^−1^ K^−1^) is the universal gas constant and *T* (K) the temperature.

At the fruit surface, the following boundary condition was assumed:

$$-D_{i}\frac{\partial C_{i}}{\partial n}=h_{i}\left(C_{i}-C_{i,\infty }\right)$$ (3)

with *n* the outward normal to the surface; the index ∞ referring to the gas concentration of the ambient atmosphere; and *h*_*i*_ the skin permeability for gas *i* (m s^−1^) (see Table 1).

**Table 1.** Parameters of the respiration diffusion model

Physical parameters	O_2_	CO_2_
Diffusivity of cortex tissue (m^2^ s^−1^)	(1.32 ± 0.39)×10^–8*a*^	(2.12 ± 0.34)×10^–8*a*^
Skin permeability (m s^−1^)	6.74 × 10^–7*b*^	10.2 × 10^–7*b*^
Respiration parameters		
$K_{m,O_{2}}$ (kPa)	0.17^*c*^	
$K_{m,f,CO_{2}}$ (kPa)		2.4 × 10^–2*c*^
$K_{mn,CO_{2}}$ (kPa)		66.4^*d*^
*r*_q,ox_		0.77 ± 0.03^*e*^
Maximal O_2_ consumption rate $V_{m,O_{2}}$ (µmol m^−3^ s^−1^)	See Table 2	
Maximal CO_2_ fermentative production rate $V_{m,f,CO_{2}}$ (µmol m^−3^ s^−1^)		See Table 2
Parameters of dynamic adaption of $V_{m,O_{2}}$to O_2_ level at 0 °C		
*V*_R,1_ (µmol m^−3^ s^−1^)	0.34×*V*_R,2_^*f*^	
*V*_R,2_ (µmol m^−3^ s^−1^)	14.9^*f*^	
Δ*V*_R_*=V*_R,2_–*V*_R,1_ (µmol m^−3^ s^−1^)	0.66×*V*_R,2_^*f*^	
*K*_**d**_ (d^−1^)	1.3^*f*^	
*m*	2^*f*^	
*K*_H_ (kPa^−2^)	23.3^*f*^	
$V_{m,f,O_{2}}$ (µmol m^−3^ s^−1^) at 0 °C		1.7.8 ± 1.7^*g*^

^*a*^ Ho *et al*. (2015).

^*b*^ Value computed from simulated 3-D microscale of epidermis tissue.

^*c*^ Ho *et al.* (2013).

^*d*^ Ho *et al.* (2008).

^*e*^ Value was calculated from the ratio of measured $R_{CO_{2}}$ to $R_{O_{2}}$ in ambient air at 20 °C (experiment A).

^*f*^Value was estimated from the experimental data (experiment B and C) at 0 °C (see more details in Table 3).

^*g*^ Value was calculated from the measurement (experiment A, see Table 2).

The gasses within the head space of a closed jar were assumed to be uniformly distributed given their fast diffusivities in air (typically five magnitudes higher than in fruit). Therefore, for an intact fruit placed in the closed jar, the O_2_ and CO_2_ concentration in the headspace of the jar changed in response to the respiration of the pear fruit and was modelled as follows:

$$V_{\text{air}}\frac{\partial C_{i,\infty }}{\partial t}=\int_{V_{\text{fruit}}}R_{i}dV$$ (4)

where *V*_fruit_ (m^3^) and *V*_air_ (m^3^) and are the volume of the fruit and the free air volume of the jar, respectively. The term on the right-hand side expresses the respiration of the entire fruit. Equations 1–4 were numerically solved using the finite element method (Comsol 3.5, Comsol AB, Stockholm) on a 3-D pear geometry generated by means of the shape generator (Rogge *et al.*, 2015).

### Response of respiration to the external O_2_ level at low temperature

A non-competitive inhibition model (Hertog *et al.*, 1998; Lammertyn *et al.*, 2001; Ho *et al.*, 2010*b*) was used to describe the consumption of O_2_ by respiration as formulated by Equation 5)

$$R_{O_{2}}=-\frac{V_{m{,}_{O_{2}}}\times [{\text{O}}_{2}]}{\left(K_{m,O_{2}}+[{\text{O}}_{2}]\right)\times \left(1+\frac{\left[{\text{CO}}_{\text{2}}\right]}{K_{mn,C_{O_{2}}}}\right)}$$ (5)

with $V_{m,O_{2}}$ (µmol m^−3^ s^−1^) the maximum oxygen consumption rate, [O_2_] (µmol m^−3^) the O_2_ concentration, [CO_2_] (µmol m^−3^) the CO_2_ concentration, $K_{m,O_{2}}$ (µmol m^−3^) the Michaelis–Menten constant for O_2_ consumption, $K_{mn,CO_{2}}$ (µmol m^−3^) the Michaelis–Menten constant for non-competitive CO_2_ inhibition, and $R_{O_{2}}$ (µmol m^−3^ s^−1^) the O_2_ consumption rate of the tissue.

The equation for the production rate of CO_2_ consists of an oxidative respiration part and a fermentative part (Peppelenbos and van’t Leven, 1996; Ho *et al.*, 2010*b*).

$$R_{CO_{2}}=-r_{q,ox}\times R_{O_{2}}+\frac{V_{m,f,CO_{2}^{}}}{\left(1+\frac{\left[{\text{O}}_{2}\right]}{K_{m,f,O_{2}}}\right)}$$ (6)

with $V_{m,f,CO_{2}}$ (µmol m^−3^ s^−1^) the maximum fermentative CO_2_ production rate, $K_{m,f,O_{2}}$ (µmol m^−3^) the Michaelis–Menten constant of O_2_ inhibition on fermentative CO_2_ production, *r*_q,ox_ the respiration quotient at high O_2_ partial pressure, and $R_{CO_{2}}$ (µmol m^−3^ s^−1^) the CO_2_ production rate of the sample.

**Fig. 1.**
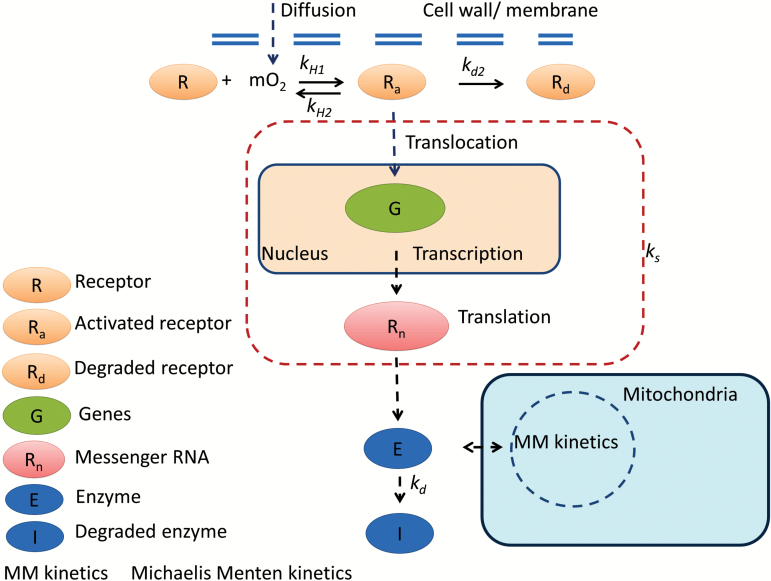
Proposed response of receptor, enzyme, and respiration to O_2_ level. The receptor in a pear cell was assumed to be activated by O_2_, and subsequently to trigger a biochemical chain involving transcription and translation steps, resulting in the final level of the enzyme *E*. A change in level of the enzyme *E* in response to the activated receptor was characterized by a lumped synthesis rate *k*_s_ agglomerating multiple conversion steps in a signal transduction cascade. Solid arrows represent direct conversions, while the dashed arrows indicate more general pathway interactions containing multiple conversion steps. Reaction equations are derived and shown in Supplementary Protocol S1 and Supplementary Fig. S1.

To account for a regulatory mechanism that would adapt the maximal respiration rate $V_{m,O_{2}}$ in response to changing O_2_ levels, we assumed that a sensor would be activated by O_2_, resulting in a signal transduction cascade that eventually would change the amount of enzymes involved in respiration (Fig. 1). A decrease of the O_2_ level would alter $V_{m,O_{2}}$due to adjustment of the balance between enzyme synthesis and degradation according to Supplementary Protocol S1 and Supplementary Fig. S1

$$\frac{\partial V_{m,O_{2}}}{\partial t}=k_{d}\times \left(V_{\text{R}}-V_{{\text{m,O}}_{\text{2}}}\right)$$ (7)

$$V_{\text{R}}=V_{\text{R},1}+\frac{\left(V_{\text{R,2}}-V_{\text{R,1}}\right)\times {\left[{\text{O}}_{2}\right]}_{}^{\text{m}}}{K_{\text{H}}+{\left[{\text{O}}_{2}\right]}_{}^{\text{m}}}$$ (8)

where *k*_d_ (d^−1^) is the rate of the response of $V_{m,O_{2}}$to changing O_2_ levels; *K*_H_ is the sensitivity of $V_{m,O_{2}}$to O_2_; and *m* is the number of O_2_ molecules aggregating one signal molecule. *V*_R,2_ in Equation 8 is the maximal O_2_ consumption rate in the presence of O_2_, while *V*_R,1_ is a base affinity for O_2_; *V*_R_ is the maximal O_2_ consumption rate at a steady O_2_ condition. Δ*V*_R_*=V*_R,2_*–V*_R,1_ is the amplitude of regulation of the maximal respiration rate by O_2_. Equations 7 and 8 imply that $V_{m,O_{2}}$may vary depending on the O_2_ concentration in a hyperbolic way between *V*_R,1_ and *V*_R,2_, and that this change is not abrupt but according to an exponential (first-order) response. Equations 1–6 will further be called the ‘gas exchange model’ and Equations 1–8 the ‘adapted gas exchange model’.

The maximal O_2_ consumption rate $V_{m,O_{2}}$and the maximal fermentative CO_2_ production rate $V_{m,f,CO_{2}}$ are temperature dependent and were assumed to follow Arrhenius’s law (Hertog *et al.*, 1998) (see Supplementary Protocol S2).

### Model parameters

The apparent O_2_ and CO_2_ diffusivities of tissue were computed from microscale simulations in small cubical samples obtained from synchrotron radiation X-ray tomography images as described by Ho *et al.* (2015) (see Table 1). Diffusivities of the tissue depend not only on the porosity but also on the degree of connectivity of the pores since diffusion in the gas phase of a gas was mainly through the connected pores but not through the dead pores (unconnected pores). We did not need to differentiate between the cortex and the ovary ground tissue since the ovary ground tissue is located in the fruit core and its size is much smaller compared with the fruit size.

The Michaelis–Menten constant, which is a ratio of rate constants, would be expected to be relatively independent of temperature (Hertog *et al.*, 1998). The values of $K_{m,O_{2}}$ and _$K_{m,f,O_{2}}$_ were therefore assumed to be independent of temperature and are taken from Ho *et al.* (2013) (Table 1).

$V_{m,f,CO_{2}}$was obtained from the CO_2_ production rate measured at 0 kPa O_2_ (data of experiment A). The maximal O_2_ consumption rate *V*_R,2_ was obtained from the O_2_ consumption rate measured at 21 kPa O_2_ (data of experiment A) in which the O_2_ consumption rate was assumed to be saturated. The parameters *k*_d_, *K*_H_, and *V*_R,1_ of the adapted gas exchange model were estimated by minimizing the squared difference between O_2_ the consumption rates predicted by Equations 1–8 and those measured from experiments B and C using a non-linear least squares estimation program written in Matlab (The Mathworks, Inc., USA) integrated with Comsol Multiphysics v. 3.5. In the estimation, a 2-D axisymmetrical model of the pear was implemented. Note that simulated results obtained from the 2-D axisymmetrical geometry were similar to those obtained from the 3-D geometry. However, the model with the 2-D axisymmetrical geometry had a low number of degree of freedoms, hence the computational time of the estimation was reduced considerably. The effect of temperature on the respiration parameters was not considered in the estimation since experiments B and C were carried out at constant temperature (0 °C).

The data of experiment D were used for validation purposes only.

### Factors affecting fruit respiration

The validation experiment was carried out in a closed system. Response of respiration as a function of the O_2_ level was in fact affected by three main factors, namely accumulation of CO_2_ during respiration, regulation of the respiratory metabolism, and O_2_ diffusion limitation. A series of simulations was carried out to analyse the relative contribution of these factors to decreasing the fruit respiration rate.

The O_2_ level inside a closed jar with an entire fruit was simulated. From this, the actual O_2_ consumption rate $R_{{\text{O}}_{2}\text{,f}}$ was computed as a function of the (decreasing) O_2_ level where the rate of the response of $V_{m,O_{2}}$to changing O_2_ levels follows Equations 7 and 8. Next, a reference O_2_ consumption rate $R_{{\text{O}}_{2}\text{,nC}}$ was computed assuming no CO_2_ inhibition ($K_{mn,CO_{2}}=+\infty$in Equation 5). Another reference O_2_ consumption rate $R_{{\text{O}}_{2}\text{,nDR}}$ was computed as a function of time assuming a constant $V_{m,O_{2}}$.

The relative effect of CO_2_ inhibition $f_{CO_{2}}$ on the O_2_ consumption rate due to CO_2_ accumulation was then calculated from:

$$f_{{\text{CO}}_{\text{2}}}=\frac{R_{{\text{O}}_{2}\text{,nC}}-R_{{\text{O}}_{2}\text{,f}}}{\max\left(R_{{\text{O}}_{2}}\right)}$$ (9)

where $\max\left(R_{O_{2}}\right)=\max\left(R_{O_{2},f},R_{O_{2},nC},R_{O_{2},nDR}\right)$ is the maximum O_2_ consumption rate of the fruit during the entire simulation for normalization purposes.

The relative effect of down-regulation *f*_DR_ was computed from:

$$f_{\text{DR}}=\frac{R_{{\text{O}}_{2}\text{,nDR}}-R_{{\text{O}}_{2}\text{,f}}}{\max\left(R_{{\text{O}}_{2}}\right)}$$ (10)

The relative effect of the remaining diffusion limitation *f*_DL_ on the O_2_ consumption rate was calculated from:

$$f_{\text{DL}}=1-f_{{\text{CO}}_{\text{2}}}-f_{\text{DR}}-R_{{\text{O}}_{2}\text{,f}}$$ (11)

## Results

### Temperature dependency of respiration capacity

**Fig. 2.**
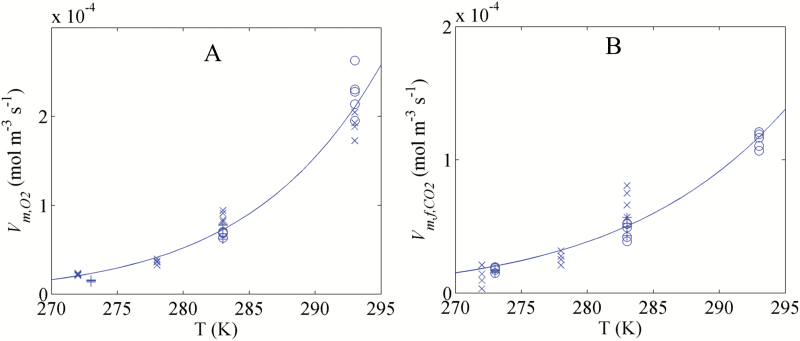
Temperature dependency of the maximal O_2_ consumption rate $V_{m,O_{2}}$ (A) and the maximal fermentative CO_2_ production rate $V_{m,f,CO_{2}}$(B). Symbols indicate the measurements, while lines present the Arrhenius model. Symbols (+), (open circles) and (×) indicate the measurements in 2010, 2014, and 2016, respectively.

The maximal O_2_ consumption rate $V_{m,O_{2}}$and the maximal fermentative CO_2_ production rate $V_{m,f,CO_{2}}$ measured at different temperatures are shown in Table 2. Temperature had a significant effect on the pear respiration. Because of biological variability, $V_{m,O_{2}}$ and $V_{m,f,CO_{2}}$ were found to vary from batch to batch, depending on fruit maturity and season. For example, a difference as great as 29 % was found for $V_{m,O_{2}}$ measured at 10 °C between season 2010 (87.8 ± 5.9 µmol m^−3^ s^−1^ and season 2016 (67.9±.3.0 µmol m^−3^ s^−1^). The temperature dependency of $V_{m,O_{2}}$and $V_{m,f,CO_{2}}$was further modelled using Arrhenius’ equations (Supplementary Protocol S2). The results are shown in Fig. 2. The maximal respiration rates were significantly affected by the temperature since the estimated values of the activation rates $E_{{\text{a,V}}_{{\text{m,O}}_{\text{2}}}}$ and $E_{{\text{a,V}}_{{\text{m,f,CO}}_{\text{2}}}}$ were equal to 73.4 ± 3.8 kJ mol^−1^ and 58.5 ± 3.3 kJ mol^−1^, respectively. $V_{m,O_{2}}$ and $V_{m,f,CO_{2}}$ exponentially increased with increasing temperature. The predicted $V_{m,O_{2}}$ values at 0 °C and 5 °C were 16% and 66% larger than those measured at the same temperatures, respectively. In addition, some variability between the measured $V_{m,O_{2}}$ and $V_{m,f,CO_{2}}$ in different seasons was observed. Therefore, in the model for a particular season, the input maximal rates were taken from the measured data in the same season to compensate for seasonal differences that otherwise would obscure the effects of changing O_2_ levels in relation to temperature that we were interested in.

**Table 2.** Measured maximal O_2_ consumption $V_{m,O_{2}}$ and maximal CO_2_ production rates during different seasons

Season	Temperature (°C)	$V_{m,O_{2}}$ (µmol m^−3^ s^−1^)	$V_{m,f,CO_{2}}$ (µmol m^−3^ s^−1^)
2010	20	230 ± 25	114 ± 6
	10	67.9 ± 3.0	46.9 ± 6.1
	0	–	17.8 ± 1.7
2014	10	71.4 ± 8.0	50.4 ± 5.8
	0	14.9 ± 0.9	16.6 ± 10.2
2016	20	190 ± 13	–
	10	87.8 ± 5.9	69.6 ± 10.4
	5	36.2 ± 2.9	26.3 ± 4.5
	-1	22.1 ± 1.0	12.0 ± 7.6

**Table 3.** Estimated parameters of response of respiration to external O_2_ level at low temperature

Parameters	*m*=2
*K*_d_	1.30 ± 0.23 (d^−1^)
*K*_H_	23.3 ± 5.3 (kPa^−2^)
*V*_R,1_	(0.34 ± 0.06)×*V*_R,2_ (µmol m^−3^ s^−1^)
*R* ^2^_adj_	0.77

### Diffusion limitation affects respiration of intact fruit at high temperature

**Fig. 3.**
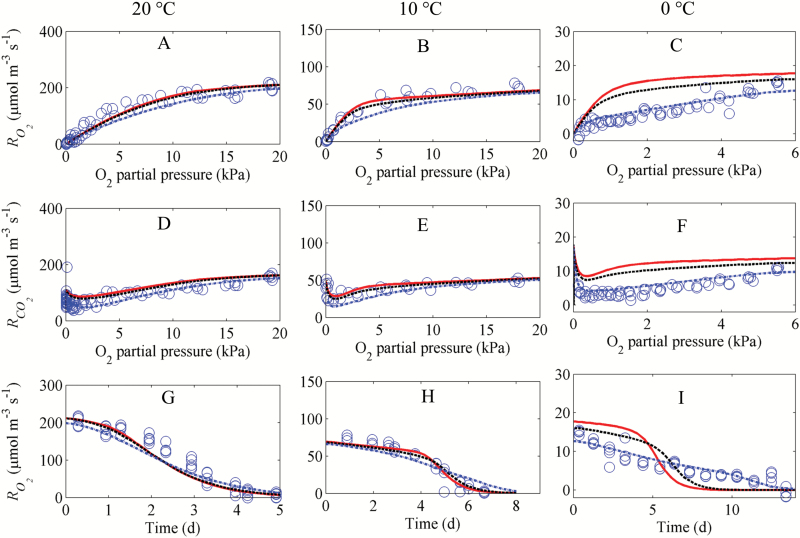
Respiration rate of intact pear fruit as a function of the O_2_ concentration at 20 °C (A, D), 10 °C (B, E), and 0 °C (C, F), and as a function of time at the same temperatures (G– I). $R_{O_{2}}$ and $R_{CO_{2}}$ are the O_2_ consumption rate and CO_2_ production rate, respectively. Open circles indicate measurements (experiment D). Solid lines (—), dashed lines ( – –), and dotted lines (⋅⋅⋅) correspond to simulations with an assumed Δ*V*/*V*_R,2_ of 0, 0.21, and 0.66, respectively. The ratio Δ*V*/*V*_R,2_ represents the amplitude of the regulation of maximal respiration rate by O_2_ (see Supplementary Protocols S1, S3 for its derivation). The maximal O_2_ consumption rate *V*_R,2_ at 20, 10, and 0 °C was measured at 21 kPa O_2_, 0 kPa CO_2_, and was equal to 230, 71.4, and 18.4 µmol m^−3^ s^−1^, respectively.

**Fig. 4.**
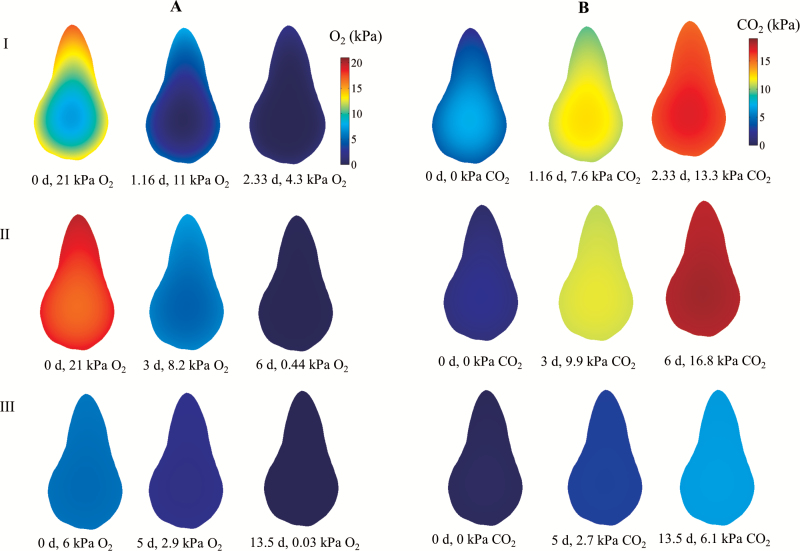
Simulated O_2_ (A) and CO_2_ (B) partial pressure inside pear fruit in a closed jar at 20 °C (I), 10 °C (II), and 0 °C (III) and different times. The initial atmosphere composition was 21 kPa O_2_, 0 kPa CO_2_, and 79 kPa N_2_ (I and II), and 6 kPa O_2_, 0 kPa CO_2_, and 94 kPa N_2_ (III). The contour graphs in (A) and (B) represent the O_2_ and CO_2_ partial pressures inside the pear, respectively. The ratio Δ*V*/*V*_R,2_ was set to 0.21, 0.21, and 0.66 at 20, 10, and 0 °C, respectively.

In the next step, the gas exchange model was used to evaluate whether respiration was diffusion limited by comparing simulated and measured values of respiration at 10 °C and 20 °C (Fig. 3) assuming a constant maximal O_2_ consumption rate ($V_{m,O_{2}}$=*V*_R,2_ Initially the O_2_ concentration in the closed jar was 21 kPa, but, as O_2_ was consumed by respiration, the O_2_ concentration and thus the O_2_ consumption rate of the fruit $R_{O_{2}}$ decreased with time. The O_2_ gradient inside the pear changed from steep in the beginning to shallow at the end of the experiment (Fig. 4). There was good agreement between simulated and measured values of respiration rates.

The simulation results suggest that at 20 °C the large respiration rate caused a rapid depletion of O_2_ towards the centre of the fruit. In combination with the diffusion resistance of the fruit cortex and skin tissue, this caused a steep O_2_ gradient inside the fruit. As a consequence, the limited O_2_ availability in the centre of the fruit reduced the local and thus also the overall respiration rate (Fig. 3A, D).

At 10 °C, the respiration rate was considerably smaller (Fig. 3B, E). Since the diffusivity of O_2_ and CO_2_ is only slightly affected by temperature, the relative rate of O_2_ transport compared with consumption was higher than at 20 °C, and the O_2_ (and CO_2_) gradient was more shallow (Fig. 4). The O_2_ concentration in the jar at which the O_2_ consumption rates decreased to half their maximal values was equal to 1.8 kPa and 4.9 kPa at 10 °C and 20 °C, respectively. The duration of the experiments at 20 °C and 10 °C was 5 d and 7 d, respectively (Fig. 3G, H).

To evaluate whether the maximal respiration rate varied during the experiment, respiration measurements were carried out at 21 kPa O_2_, 0 kPa CO_2_, and 10 °C on four consecutive days. The respiration rate after 3 d increased slightly by 3%, but was not significantly different from that at day 1 (Supplementary Fig. S2).

### Down-regulation of respiration at low temperature

At 0 °C the respiration rate was very low (one order of magnitude lower than respiration at 20 °C), and it would take a considerable amount of time to deplete the O_2_ in the jar. We therefore started the experiment at an O_2_ concentration of 7 kPa. As the rate of O_2_ diffusion was now much larger than that of O_2_ consumption, the O_2_ concentration profile was now almost uniform and there were hardly any gradients (Fig. 4). The rate-limiting enzyme of respiration is believed to be COX (Armstrong and Beckett, 2011*a*), and we thus expected a Michaelis–Menten-like behaviour with a saturation O_2_ consumption rate at an O_2_ level larger than the *K*_m_ of COX. Surprisingly, the measurements showed a clear linear decrease of both the O_2_ consumption rate and the CO_2_ production rate with decreasing O_2_ levels until 1 kPa. (Fig. 3C). The ratio of $R_{CO_{2}}$ to $R_{O_{2}}$was >1 at an O_2_ level lower than 0.5 kPa (Fig. 3F). By assuming a constant $V_{m,O_{2}}$ the gas exchange model predicted a Michaelis–Menten-like overall respiration rate $R_{O_{2}}$ that saturated at sufficiently large O_2_ concentrations, which was not consistent with the measurements (Fig. 3C, F). We thus modified our model to incorporate an adaption of $V_{m,O_{2}}$to the O_2_ level in the jar.

### Dynamic adaption of $V_{m,O_{2}}$to O_2_ level at low temperature

**Fig. 5.**
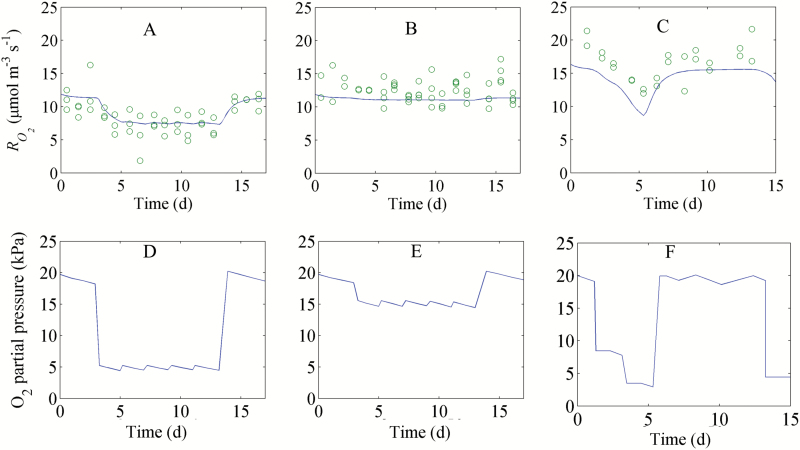
Dynamic response of the O_2_ consumption rate $R_{O_{2}}$ to changing O_2_ levels and time at 0 °C (experiment B). (A–C) and (D–F) represent the $R_{O_{2}}$ and external O_2_ level as a function of time, respectively. Open circles and solid lines (—) indicate measurements and model predictions, respectively.

**Fig. 6.**
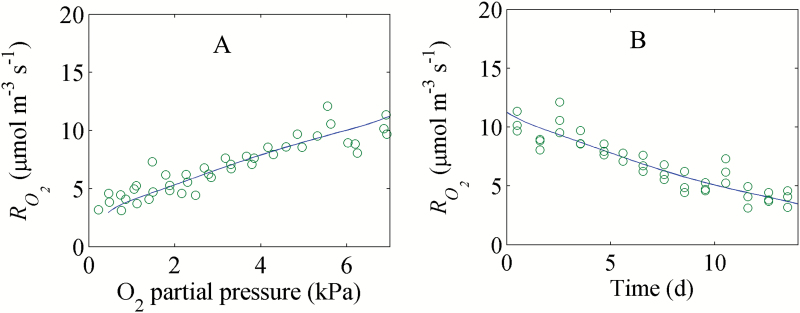
Response of the $R_{O_{2}}$ of intact pear fruit to O_2_ (A) and time (B) (experiment C). Open circles and solid lines (—) indicate measurements and model predictions, respectively.

In a next step we estimated the parameters *k*_d_, *K*_H_, and *V*_R,1_ of Equations 7 and 8 using the combined data of experiment B and C (Supplementary Table S1). At 0 °C, *V*_R,2_ in Equation 8 was set equal to the measured O_2_ consumption rate at 21 kPa O_2_, 0 kPa CO_2_, and 79 kPa N_2_. We observed that the O_2_ consumption rate at 21 kPa O_2_ and 0 kPa CO_2_ was not constant but decreased during long-term storage at –1 °C (Supplementary Fig. S3) but at a rather slow pace (–3.64 × 10^–2^ µmol m^−3^ s^−1^ d^−1^). Hence, *V*_R,2_ was set equal to the measured O_2_ consumption rate at the initial time of the experiment, assuming it to be constant for the duration of the simulated storage period of 15 d (Supplementary Fig. S3). A good agreement between the fitted respiration rates and corresponding measurements was observed (Figs 5, 6). The change of $R_{O_{2}}$ with an abrupt decrease of the O_2_ level from 20 kPa to 5 kPa O_2_ (Fig. 5A, D) was large; when O_2_ decreased from 20 kPa to 15 kPa O_2_, it was hardly visible (Fig. 5B, E). We found that the adapted respiration model with *m* equal to 2 gave a better fit to the observed data than that with *m* equal to 1 (Supplementary Figs S4, S5). *R*^2^, a criterion for the goodness of fit (see definition in Supplementary Protocol S4), was 0.677 and 0.753 for the adapted respiration model with *m* equal to 1 and 2, respectively. Therefore, only estimated parameters with *m* equal to 2 were considered. The estimated values of *k*_d_ and *K*_H_ were 1.30 ± 0.23 d^−1^ and 23.3 ± 5.3 kPa^2^, respectively (Table 2). The estimated value of *K*_H_ implied that $V_{m,O_{2}}$ reduced to half of *V*_R,2_ at a constant O_2_ level of 4.8 kPa (Supplementary Fig. S6A). The estimated value of *k*_d_ suggested that the time for$V_{m,O_{2}}$–*V*_R_ to decrease by 37% in response to a sudden drop in the O_2_ concentration was 0.77 d (Supplementary Fig. S6B). The estimated value of *V*_R,1_ was 0.34 ± 0.06×*V*_R,2_. All in all, these results indicate that even at O_2_ concentrations much larger than the *K*_m_ of COX, the respiration rate of the fruit is reduced, presumably due to down-regulation of key enzymes of the respiration pathway.

We also tested the alternative hypothesis that $K_{m,O_{2}}$ varied at low temperature but $V_{m,O_{2}}$ remained constant. The estimated value of $K_{m,O_{2}}$was 2.04 ± 0.01 kPa, which was much larger than that of pear cell protoplasts and isolated mitochondria. Since the *R*^2^ of this model (0.668) was lower than that of the adapted respiration model with *m* equal to 2 (0.753) and the fit was also worse than that of the adapted respiration model with *m* equal to 2 (Supplementary Figs S7, S8), we rejected this alternative hypothesis.

### Adaption of respiration in response to O_2_ level at different temperatures

We further tested the hypothesis that while adaption of respiration with O_2_ levels was considerably high at low temperature, it was relatively insignificant at high temperature. As can be seen in our model analysis, Δ*V* at 0 °C was 66% of the total maximal respiration rate *V*_R,2_. We further simulated different responses of respiration to O_2_ level with Δ*V*/*V*_R,2_ of 0, 0.21, and 0.66, respectively. At 20 °C and 10 °C, the simulated results were comparable with the measured values when Δ*V*/*V*_R,2_ was low (0 or 0.21) (Fig. 3). In contrast, at 0 °C, the model fitted the measured data best for Δ*V*/*V*_R,2_ equal to 0.66. Replicate measurements of the respiration rate in response to different O_2_ levels were additionally carried out in 2016 at 5, 10, and 20 °C (Supplementary Fig. S9). Again the simulated O_2_ and CO_2_ consumption rates at 10 °C and 20 °C with Δ*V*/*V*_R,2_ equal to 0 or 0.21 fitted the data well (Supplementary Fig. S9A–D) while at 5 °C a Δ*V*/*V*_R,2_ of 0.66 gave the best fit (Supplementary Fig. S9E, F). Our simulation results confirmed that down-regulation was temperature dependent and significant at low temperature.

### Factors affecting fruit respiration

**Fig. 7.**
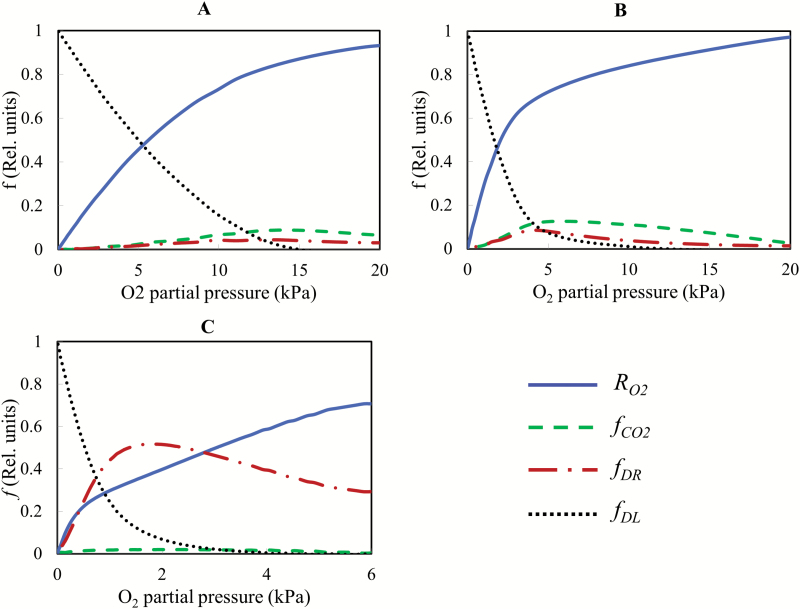
Factors affecting the O_2_ consumption rate as a function of the O_2_ level. (A–C) Temperatures of 20, 10, and 0 °C. $R_{O_{2}}$ is the O_2_ consumption rate, while $f_{CO_{2}}$*f*_DR_, and *f*_DL_ are the relative effect of CO_2_ inhibition, down-regulation, and diffusion limitations on the O_2_ consumption rate, respectively. Δ*V*/*V*_R,2_ was set to 0.21, 0.21, and 0.66 at 20, 10, and 0 °C, respectively.

The relative importance of the different factors affecting the respiration rate under decreasing O_2_ levels is shown in Fig. 7. At 20 °C, respiration was rapidly reduced by diffusion limitations at a high respiration rate when the O_2_ concentration decreased. The effect was found to increase predominantly when the O_2_ partial pressure decreased to <12 kPa (Fig. 7A). At 10 °C, respiration was slightly reduced by the accumulating CO_2_ concentration when the experiment evolved and the O_2_ concentration decreased. When the O_2_ partial pressure decreased below 4 kPa, diffusion limitations caused a progressively steep decline of the O_2_ consumption curve. Note that at this stage, although the accumulated CO_2_ level was high, CO_2_ inhibition of respiration was much smaller than that caused by diffusion limitation (Fig. 7B).

At 0 °C, the inhibition of respiration by CO_2_ as shown in Fig. 7C was less profound due to the limited accumulation of CO_2_. Also, the simulation results showed that diffusion limitations at O_2_ levels >2 kPa did not affect respiration much. A bi-linear decrease was found. The first decrease was the adaptive response of the respiration rate at the O_2_ level >2 kPa probably due to down-regulation. When the O_2_ level was <2 kPa, however, the rate of O_2_ diffusion through tissue became more predominantly limiting as the decline of the O_2_ consumption rate became sharper until it reached zero.

## Discussion

### Potential of gas exchange model in a systematic study of respiration response to O_2_ level

Armstrong and Beckett (2011*a*) have shown the effect of O_2_ diffusion on respiration of roots using a reaction–diffusion model on a multicylindrical geometry. These authors did not explicitly determine diffusion coefficients. To better understand the response of respiration to O_2_ level, we analysed the respiration behaviour by means of a reaction–diffusion model that incorporated more detailed Michaelis–Menten kinetics for O_2_ and CO_2_ consumption and production, respectively, and that accounted for CO_2_ inhibition effects. The corresponding diffusion coefficients were calculated by means of a microscale model following Ho *et al.* (2016). While earlier modelling work did show an obvious internal concentration gradient in pear at low temperature (Ho *et al.*, 2008), this did not become visible in the current work. The gradients predicted before came from an underestimation of the experimentally measured diffusivities due to artefacts induced by cutting of the samples, inundating the intercellular spaces by leaking of the cell content (Ho *et al.*, 2016). The current model predictions are more reliable as they are based on the improved diffusion properties determined from simulations at the microscale based on 3-D synchrotron microtomography images (Ho *et al.*, 2016).

Simulations showed that at above 10 °C, the overall respiratory activity of the fruit was predicted well by a gas diffusion model incorporating Michaelis–Menten kinetics to describe respiration, suggesting that under these conditions respiration is mainly controlled by diffusion limitations. Similar results were reported by Armstrong and Beckett (2011*a*) for root pieces. While respiratory down-regulation was not clearly found in our measurement and simulation results at high temperature, such an effect could have been annihilated by an increase of $V_{m,O_{2}}$ by fruit ripening during the course of the experiment. However, this was not the case as the measurements showed that $V_{m,O_{2}}$ did not significantly change during the experimental period (Supplementary Fig. S2). The observed decrease of respiration was therefore mainly due to diffusion limitation and the inhibition effect of accumulated CO_2_ (Fig. 7A, B).

The value of *r*_q_ was equal to 0.77, indicating that the measured CO_2_ production rate was lower than the measured O_2_ consumption rate. Note that *r*_q_ for an ideal respiration with carbohydrate substrate is considered equal to 1, and CO_2_ solubilization might cause undercalculation of the CO_2_ production rate. Our simulations with the input parameter *r*_q_ equal to 1 predicted much larger CO_2_ production rates compared with those of the measurements. The CO_2_ production rate profiles predicted with parameter *r*_q_ equal to 0.77 were well comparable with the measured profiles (Fig. 3D–F). The simulations indicated that the CO_2_ production rate is likely to be unaffected by CO_2_ solubility.

### Down-regulation may explain the response of respiration to O_2_ level at low temperature

The conventional Michaelis–Menten-like model for gas exchange assumes that the respiration rate of plant cells would already saturate at an O_2_ level as low as 1.5 kPa in the tissue. When we measured respiration rates at 0 °C (Fig. 3C, F) and 5 °C (Supplementary Fig. S9E, F) to minimize the effect of O_2_ diffusion, we found that the O_2_ consumption rate was considerably reduced in response to decreasing O_2_ levels well above 1.5 kPa (nine times the *K*_m_ of tissue). In the experiments, the cooling capacity of our experimental cold rooms was sufficiently large to decrease the temperature from 20 °C to 0 or 5 °C within hours and keep the fruit at constant and almost uniform temperature (Supplementary Protocol S5; Supplementary Fig. S10). Note that *K*_m_ represents the O_2_ concentration at which the respiration rate reaches half of its maximum value (Armstrong and Beckett, 2011*a*). *K*_m_ might be temperature dependent, similar to the maximum respiration rate (Kruse *et al.*, 2011). However, Hertog *et al.* (1998) proposed that *K*_m_, being a ratio of rate constants, was relatively independent of temperature when the activation energies of individual rate constants were similar. The value of *K*_m_ is 0.17 kPa O_2_ for tissue (Ho *et al.*, 2013), 0.14 kPa O_2_ for cell protoplasts (Lammertyn *et al.*, 2001), and 0.10–1 µM (~4.5 × 10^–3^–4.5 × 10^–2^ kPa) for COX [0.10–0.12 µM (~4.5 × 10^–3^–5 × 10^–3^ kPa), Rawsthorne and Larue (1986); 1 µM (~4.5 × 10^–2^ kPa), Taiz and Zeiger (1993), and 0.14 μM (~6 × 10^–3^ kPa), Millar *et al.* (1994)]. *K*_m_ described in the model was much larger than that obtained from isolated mitochondria since *K*_m_ for tissue accounted for diffusion barriers through the cell wall, cell membrane, and within the cytosol. Simulations with a two-compartment model (core and cortex) and different combinations of O_2_ diffusivities and *V*_max_ values were also carried out (Supplementary Fig. S11). Respiration was assumed to follow conventional Michaelis–Menten kinetics without a regulatory mechanism. While at 10 °C the model fitted the data well, this was not the case at 0 °C for any of the aforementioned parameter combinations. The experimental data contradicted simulation results obtained with the gas exchange model incorporating conventional Michaelis–Menten-based respiration kinetics, suggesting an additional reduction in respiration rate beyond the substrate effects already accounted for. When we modified the respiration kinetics to allow for $V_{m,O_{2}}$ to change as a function of the O_2_ level rather than keeping $V_{m,O_{2}}$ constant, the simulations fitted the measurements well (Fig. 3C, E, F; Supplementary Fig. S9E, F). This indicated that additional regulatory effects of the respiration pathways are likely to occur. The dynamics of regulatory and signalling pathways in the cell were modelled by reaction kinetics at the transcriptome level. We assumed that an O_2_ signal could modulate the biosynthesis of respiratory enzymes in the cell through activation of an O_2_ receptor. The response of the maximal respiration rate was proportional to the change of amount of enzymes involving in the respiration. We assumed that a decrease of the O_2_ level would alter the maximal respiration rate due to adjustment of the balance between enzyme synthesis and degradation (see Fig. 1; Supplementary Protocol S1; Supplementary Fig. S1). So, fundamentally, the model allows a bidirectional change in enzyme activity. We observed a relatively slow adaption to changing O_2_ levels with an estimated *k*_d_ of 1.30 d^−1^. Our results showed that the response of $V_{m,O_{2}}$to changing O_2_ levels was more sensitive at low O_2_ than at high O_2_ levels (see Figs 3, 4; Supplementary Figs S9, S12).

Zabalza *et al.* (2009) found that the respiratory demand in pea root was 300 nmol g^−1^ min^−1^ O_2_ (equivalent to 5 × 10^4^ µmol m^−3^ s^−1^) for pea root at 25 °C. For barley root at 25 °C, the respiratory demand was observed to be 100 µmol g^−1^ h^−1^ O_2_ (equivalent to 2.7 × 10^5^ µmol m^−3^ s^−1^) (Gupta *et al.*, 2009). These values are considerably larger than that of pear fruit measured in this study (230 µmol m^−3^ s^−1^ at 20 °C). This difference is due to the fact that mature but pre-climacteric pear fruit are much less metabolically active as compared with roots that are actively involved in uptake processes. Respiratory down-regulation in plant tissues has been suggested by Gupta *et al.* (2009) and Zabalza *et al.* (2009). Zabalza *et al.* (2009) observed a slow but linear decrease of the respiratory rate of roots of pea and Arabidopsis with decreasing O_2_ levels until ~4 kPa, below which it steeply declined. However, the respiratory demand of roots of pea and Arabidopsis in the experiments performed by these authors was more than an order of magnitude larger than the pear respiration performed at 20 °C in this study, resulting in scavenging of oxygen from their system being completel in <2 h. Since down-regulation might require long exposure at specific O_2_ levels, a change of respiration to O_2_ levels has been alternatively suggested by substantial diffusion limitation on O_2_ supply when O_2_ respiratory demand was high (Armstrong and Beckett, 2011*a*). At low temperature due to low respiration demand, the adaptive response of the respiration rate to O_2_ levels was shown to be due to down-regulation rather than diffusion limitation on O_2_ supply (Figs 3C, E, F, 4; Supplementary Figs S13, S14). This has implications with respect to commercial storage of pear fruit under hypoxic conditions [‘controlled atmosphere (CA) storage’]. Abruptly and drastically changing the O_2_ level is known to cause browning and cavity formation in pear, probably because respiration may consume most O_2_ in the centre of the pear (Verlinden *et al.*, 2002). This may create near anoxic conditions initiating a chain of events eventually causing the symptoms of the disorder. Adaption of the fruit to low O_2_ levels by reducing the respiration rate would eventually result in less severe O_2_ concentrations in the centre of the fruit and a reduction in the symptoms. This procedure is in fact applied in practice and may be further optimized. While the model was developed using ‘Conference’ pear data, it should be extended to other pear cultivars or to ‘Conference’ pears grown under different agronomic/climate conditions that may well affect both fruit respiration and microstructural properties. Note that the gas transport model that was used herein assumes that gas transport properties are uniform and isotropic, and that the respiration kinetics do not depend on position. Future research should incorporate more realistic features into the model and investigate their effect on gas transport.

### AOX might play a role in regulation of respiration

The alternative oxidase (AOX) has been proposed to play a role in adaption of respiration to O_2_ level within mitochondria (Szal *et al.*, 2003; McDonald and Vanlerberghe, 2006; Gupta *et al.*, 2009). At a short-term temperature change from 17 °C to 36 °C, the ratio of alternative respiration to total respiration was reported to be relatively constant and ~0.21–0.30 for different leaves of *Nigella sativa*, *Cucurbita pepo*, and *Vicia faba* (Macfarlane *et al.*, 2009). However, partitioning of electrons via the alternative respiration pathway has been shown to be increased after long-term cold acclimation in some species (Gonzalez-Meler, 1999; Fung *et al.*, 2004; Sugie *et al.*, 2006). Our simulation results showed that the magnitude of regulation of respiration in response to O_2_ level was relatively low at high temperature but significantly high at low temperature. Note that we did not explicitly model distinct AOX and COX pathways. If the AOX pathway is indeed responsible for the regulatory effects by O_2_, Δ*V* can be interpreted as its capacity, while the time and O_2_ responses are lumped in the parameters *k*_d_ and *K*_H_. Assuming that at 10 °C and 20 °C the amplitude of the regulation of the respiration rate, Δ*V*, was 0.21 times the total maximal respiration rate *V*_R,2_, we found good agreement between simulation and measurements. This magnitude was similar to the partition of the AOX pathway to the total respiration at high temperature (0.21–0.30 from 17 °C to 36 °C, Macfarlane *et al.*, 2009). At 0 °C, Δ*V* was found to be 0.66 times the total maximal respiration rate *V*_R,2_. Likewise, the ratio of the AOX pathway to the total respiration for maize leaves (*Zea mays* L. cv Penjalina) growing at 25 °C was reported to be 0.25 but increased to 0.6 after 5 d at 5 °C (chilled) (Ribas-Carbo *et al.*, 2000). These results indicate that the regulation of AOX might be involved in the response of respiration to changing O_2_ levels at low temperature.

## Supplementary data

Supplementary data are available at *JXB* online.

Protocol S1. Modelling the response of $V_{m,O_{2}}$to O_2_ level.

Protocol S2. Temperature dependency of respiration capacity.

Protocol S3. Amplitude of regulation of maximal respiration rate by O_2_.

Protocol S4. Criterion for goodness of fit of the model.

Protocol S5. Heat conduction model.

Table S1. Description of data sets used in calibration and validation of model.

Fig. S1. Proposed reactions and modelled equations describing response of receptor, enzyme, and respiration to O_2_ level.

Fig. S2. O_2_ consumption rate of intact pear fruit as a function of time at 20 kPa O_2_, 0 kPa CO_2_ at 10 °C.

Fig. S3. O_2_ consumption rate of intact pear fruit as a function of time during storage of fruit at 20 kPa O_2_, 0 kPa CO_2_ at 0 °C.

Fig. S4. Dynamic response of O_2_ consumption rate $R_{O_{2}}$ to O_2_ level and time at 0 °C (experiment B).

Fig. S5. Response of $R_{O_{2}}$ of intact pear fruit to O_2_ (A) and time (B) (experiment C).

Fig. S6. (A) Steady-state modelled response of relative maximal O_2_ consumption rate to O_2_ level. (B) Change of maximal O_2_ consumption rate in response to a sudden drop of the O_2_ concentration.

Fig. S7. Comparison of fitting between the adapted respiration model (*m*=2) and the respiration model with assumption of variation of $K_{m,O_{2}}$ at 0 °C.

Fig. S8. Comparison of fitting between the adapted respiration model (*m*=2) and the respiration model with assumption of variation of $K_{m,O_{2}}$ at 0 °C.

Fig. S9. Respiration rate of intact pear fruit as a function of the O_2_ concentration at 20 °C (A, B), 10 °C (C, D), and 5 °C (E, F) harvested in season 2016.

Fig. S10. Predicted temperature of pear fruit during cooling.

Fig. S11. Simulations with a two-compartment model (core and cortex) and different combinations of diffusivities and *V*_max_ values.

Fig. S12. Steady-state modelled response of relative maximal O_2_ consumption rate ($V_{m,O_{2}}$/max$V_{m,O_{2}}$) to O_2_ level.

Fig. S13. Simulated $V_{m,O_{2}}$ of pear fruit in the closed jar at 20 °C (I), 10 °C (II), and 0 °C (III) and different times.

Fig. S14. Simulated O_2_ and CO_2_ gas partial pressure profiles from the centre to the surface along the radial direction in the closed jar at 20 °C (I), 10 °C (II), and 0 °C (III) at different times.

Supplementary Tables and Figures
