# Supplementary material for: Down-regulation of respiration in pear fruit depends on temperature
Source: J Exp Bot. 2018 Jan 31;69(8):2049–60. doi: 10.1093/jxb/ery031 (PMC6018969; doi:10.1093/jxb/ery031)
Supplement: Supplementary Tables and Figures [file ery031_suppl_suppl_table_figures.pdf]

Down-regulation of respiration in pear fruit depends on temperature

Q. T. Ho, M.L.A.T.M. Hertog, P. Verboven, A. Ambaw, S. Rogge, B. E. Verlinden and B. M. Nicolaï

SUPPLEMENTARY DATA

Protocol S1. Modelling the response of V_{m,O_2} to O_2 level

Protocol S2. Temperature dependency of respiration capacity

Protocol S3. Amplitude of regulation of maximal respiration rate by O_2

Protocol S4. Criterion for goodness of fit of the model

Protocol S5. Heat conduction model

Table S1. Description of data sets used in calibration and validation of model

Figure S1. Proposed reactions and modelled equations describing response of receptor, enzyme and respiration to O_2 level.

Figure S2. O_2 consumption rate of intact pear fruit as a function of time at 20 kPa O_2, 0 kPa CO_2 at 10 °C.

Figure S3. O_2 consumption rate of intact pear fruit as a function of time during storage of fruit at 20 kPa O_2, 0 kPa CO_2 at 0 °C.

Figure S4. Dynamic response of O_2 consumption rate (R_{O_2}) to O_2 level and time at 0 °C (experiment B).

Figure S5. Response of R_{O_2} of intact pear fruit to O_2 (A) and time (B) (experiment C).

Figure S6. (A) Steady state modelled response of relative maximal O_2 consumption rate to O_2 level. (B) Change of maximal O_2 consumption rate in response to a sudden drop of the O_2 concentration.

Figure S7. Comparison of fitting between the adapted respiration model (*m* = 2) and the respiration model with assumption of variation of K_{m,O_2} at 0 °C.

Figure S8. Comparison of fitting between the adapted respiration model (*m* = 2) and the respiration model with assumption of variation of K_{m,O_2} at 0 °C.

Figure S9. Respiration rate of intact pear fruit as a function of the O_2 concentration at 20 °C (A, B), 10 °C (C, D), and 5 °C (E, F) harvested in season 2016.

Figure S10. Predicted temperature of pear fruit during cooling.

Figure S11. Simulations with a two-compartment model (core and cortex) and different combinations of diffusivities and V_{max} values.

Figure S12. Steady state modelled response of relative maximal O_2 consumption rate (V_{m,O_2} / max(V_{m,O_2})) to O_2 level.

Figure S13. Simulated V_{m,O_2} of pear fruit in the closed jar at 20 °C (I), 10 °C (II) and 0 °C (III) and different times.

Figure S14. Simulated O_2 and CO_2 gas partial pressure profiles from the center to the surface along the radial direction in the closed jar at 20 °C (I), 10 °C (II) and 0 °C (III) at different times.

Protocol S1. Modelling the response of V_{m,O_2} to O_2 level

We assumed that a receptor in a pear cell can be activated by O_2, causing a signal transduction cascade that results in a final change in enzyme concentrations involved in respiration (Fig. S1). Change of activation of the receptor by O_2 which is assumed to be described by the Hill equation (reaction R1, Fig. S1) could be written as

$$\frac{\partial[R_a]}{\partial t} = k_{H1}\cdot[R]\cdot[\mathrm{O}_2]^m - (k_{H2}+k_{d2})\cdot[R_a] \qquad \text{(S1)}$$

where $[O_2]$ is the O_2 concentration; $[R_a]$ and $[R]$ are the concentrations of active and inactive receptors, respectively; m is the number of O_2 molecules aggregating one receptor molecule, k_{H1} and k_{H2} are the rate constants of the Hill equation; k_{d2} is the rate constant of receptor degradation; t (s) is time. Note that if $m = 1$, the Hill equation becomes a Michaelis Menten kinetics. If $m > 1$, then the binding of O_2 to the receptor causes the affinity for other O_2 molecules to increase.

Here, the activate receptor is assumed to trigger a biochemical chain involving in transcription and translation steps, resulting the final level of the enzyme E. Since quantitative kinetic parameters of the transcription and translation is unavailable, we simply assumed that change in level of the enzyme E in response to the activate receptor was characterized by a lumped synthesis rate k_s agglomerating multiple conversion steps in a signal transduction cascade. Such response of enzyme and/or protein to signal has been described by simple reaction (Tchourine *et al.*, 2014). The corresponding change of enzyme concentration E in response to level of the receptor is assumed as:

$$\frac{\partial[E]}{\partial t} = k_s\cdot[R_a]\cdot[AA] - k_d\cdot[E] \qquad \text{(S2)}$$

where $[E]$ is the concentration of enzyme initially available; k_s represents the overall rate constant of enzyme synthesis taking into account transcription and translation; k_d is the rate constant of enzyme degradation; $[AA]$ is the amount of amino acid involving in enzyme synthesis (assumed to be constant).

Respiration has been commonly described by an existing Michaelis Menten kinetics (Hertog *et al.*, 1998). In this study, we assumed that the response of the maximal respiration rate V_{m,O_2} was proportional to the change of enzymes involving in the respiration

$$\frac{\partial V_{m,O_2}}{\partial t} = k_p\frac{\partial[E]}{\partial t} \qquad \text{(S3)}$$

Where k_p is the reaction rate constant for the formation of CO_2 product (Hertog *et al.*, 1998). The Eq. (S3) can be approximated as

$$V_{m,O_2} = k_p \cdot [E] + V_{R,1} \qquad \text{(S4)}$$

Solving Eq. (S4) for [*E*], substituting into Eq. (S2) yields:

$$\frac{\partial V_{m,O_2}}{\partial t} = k_p \cdot k_s \cdot [R_a] \cdot [AA] - k_d \cdot V_{m,O_2} + k_d \cdot V_{R,1} \qquad \text{(S5)}$$

Activation of the signal can be considered at a quasi-steady state; hence Eq. (S1) is assumed to equal to zero and together with equation (S5) rearranged to obtain an expression for V_{m,O_2}:

$$\frac{\partial V_{m,O_2}}{\partial t} = k_d \cdot \left(V_R - V_{m,O_2}\right) \qquad \text{(S6)}$$

$$V_R = V_{R,1} + \frac{\left(V_{R,2} - V_{R,1}\right) \cdot [\mathrm{O}_2]^m}{K_H + [\mathrm{O}_2]^m} = V_{R,1} + \frac{\Delta V \cdot [\mathrm{O}_2]^m}{K_H + [\mathrm{O}_2]^m} \qquad \text{(S7)}$$

with $V_{R,2} = V_{R,1} + \dfrac{k_p \cdot k_s \cdot [R_T] \cdot [AA]}{k_d}$ (S8); $K_H = \dfrac{k_{H2} + k_{d2}}{k_{H1}}$ (S9);

$[R_T] = [R_a] + [R]$ (S10); $\Delta V = \dfrac{k_p \cdot k_s \cdot [R_T] \cdot [AA]}{k_d}$ (S11);

In Eq. (S7), $V_{R,1}$ is a base affinity to O_2; $V_{R,2}$ is the maximal O_2 consumption rate in the presence of O_2; ΔV represents the amplitude of the regulation of the maximal respiration rate by O_2.

Protocol S2. Temperature dependency of respiration capacity

The effect of temperature on the maximal O_2 consumption rate V_{m,O_2} and the maximal fermentative CO_2 production rate V_{m,f,CO_2} was described by Arrhenius's law (Hertog et al., 1998).

$$V_{m,O_2} = V_{m,O_2,ref} \exp\left[\frac{E_{a,V_{m,O_2}}}{R}\left(\frac{1}{T_{ref}} - \frac{1}{T}\right)\right] \qquad \text{(S12)}$$

$$V_{m,f,CO_2} = V_{m,f,CO_2,ref} \exp\left[\frac{E_{a,V_{m,f,CO_2}}}{R}\left(\frac{1}{T_{ref}} - \frac{1}{T}\right)\right] \qquad \text{(S13)}$$

where $V_{m,O_2,ref}$ (μmol m^{-3} s^{-1}) and $V_{m,f,CO_2,ref}$ (μmol m^{-3}s^{-1}) are the maximal O_2 consumption rate and maximal fermentative CO_2 production rate at T_{ref}=283°K, respectively; $E_{a,V_{m,O_2}}$ and $E_{a,V_{m,f,CO_2}}$ (kJ mol^{-1}) are the activation energies for O_2 consumption and fermentative CO_2 production; T (K) temperature; and R (8.314 J mol^{-1} K^{-1}) the universal gas constant.

Protocol S3. Amplitude of regulation of maximal respiration rate by O_2

The amplitude of regulation of maximal respiration rate by O_2, ΔV was derived from Eq. S11. In the equation, parameters k_d and k_p are the reaction rate constants of enzyme degradation and CO_2 production rate while k_s represents the overall rate constant of enzyme production. The effect of temperature on ΔV by both k_d and k_p was presumably cancelled in Eq. (S11) if they were assumed to have the same activation energy. k_s being the rate constant agglomerating multiple conversion steps including transcription and translation was assumed to be less affected by low temperature. Note that to response to low temperature stress, ribosomes might modify their translation machine to facilitate protein synthesis. For instance, the AOX amount (transcript, protein or capacity) has been reported to increase at low temperature (Fung *et al.*, 2004; Sugie *et al.*, 2006) although the respiration rate was considerably reduced. Our results indicated that ratio of $\Delta V / V_{R,2}$ was rather low at high temperature as compared to low temperature. Assuming that ΔV ranged from 0 to 0.21 fold the value of maximal respiration rate $V_{R,2}$ at 10 °C and 20 °C, the model predictions are comparable to the measurement. In contrast, at low temperature the model prediction show a good agreement with the measurement when amplitude of the regulation of respiration was high (ΔV_R equal to 0.66 fold the value of $V_{R,2}$).

Protocol S4. Criterion for goodness of fit of the model

R^2 is a statistical measure of how close the fitted model to data is. In general, the R^2 is defined as:

$$R^2 = 1 - \frac{SS_{res}}{SS_{tot}} \qquad \text{(S14)}$$

where SS_{res} and SS_{tot} are the total sum of squares of the data and the regression sum of squares, respectively. SS_{res} and SS_{tot} are defined as:

$$SS_{res} = \sum\left(y_i - f_i\right) \quad \text{(S15)}$$

$$SS_{tot} = \sum\left(y_i - \overline{y}\right) \quad \text{(S16)}$$

where y_i, $\overline{y}$ and f_i are the measured data, the mean of the measured data and the predicted value by the model, respectively.

Protocol S5. Heat conduction model

A model of heat conduction inside pear fruit was performed to predict the cooling time and temperature profile within the pear at 0 °C. Heat conduction within the pear fruit is described as follows:

$$\rho C_p \frac{\partial T}{\partial t} = \nabla \cdot k\nabla T + Q \qquad \text{(S17)}$$

where ρ (~1000 kg m^{-3}) is the fruit density, C_p (3.69 kJ kg^{-1} K^{-1}, Ramaswamy and Tung, 1981) is the heat capacity of the fruit tissue, k (0.427 W m^{-1} K^{-1}, Ramaswamy and Tung, 1981) is the heat conductivity of the fruit tissue. Q (W m^{-3}) is the heat generation. Q can be calculated from the respiration rate as follows

$$Q = -q_{O_2} R_{O_2} \qquad \text{(S18)}$$

where q_{O_2} (J mol^{-1} O_2 consumed) is the proportional constant. In aerobic conditions and when glucose is the substrate , q_{O_2} has a value of 478.3 kJ mol^{-1} O_2 consumed (Datta et al., 2005). At the fruit surface the following boundary condition was assumed:

$$-k\frac{\partial T}{\partial n} = h_m\left(T - T_\infty\right) \qquad \text{(S19)}$$

with n the outward normal to the surface; the index ∞ referring to the ambient temperature; h_m the heat transfer coefficient (W m^{-2} K^{-1}). For nature convection, value of h_m is assumed to be 5 W m^{-2} K^{-1}.

Equations S17-S19 combined with equations (1-4) were numerically solved using the finite element method (Comsol 3.5, Comsol AB, Stockholm). Simulation results showed that the pear was completely cooled from 20 °C to 0 °C after 10 h and the temperature was homogeneous throughout the fruit (temperature difference less than 0.012 °C). (Fig. S10).

Table S1. Description of data sets used in calibration and validation of model

Experiment	Date	Condition	Description
A	15/10/2010, 28/10/2010, 04/11/2014, 16/11/2016, 30/11/2016, 21/12/2016	Fruit were taken from CA storage and put at normal ambient condition at 0 °C for 3 days before starting the experiment.	Determination of the maximal O_2 consumption rate and the maximal fermentative CO_2 production rate
B1<br>B2<br>B3	26/01/2015<br>26/01/2015<br>10/11/2014	Fruit were taken from CA storage and put at normal ambient condition at 0 °C for 1 day before starting the experiment.	Estimation of parameters k_α, the time response of V_{m,O_2} to O_2 level and K_H the sensitivity of V_{m,O_2} to O_2 level at 0 °C (see Fig. 4, Fig. 5).
C	21/10/2014	Fruit were taken from CA storage and put at normal ambient condition at 0 °C for 1 day before starting the experiment.	
D1	07/11/2014	Fruit were taken from CA storage and put 7 days at normal ambient condition at 0 °C before starting the experiment.	Validation at 10 °C (see Fig. 1)
D2	15/09/2014	Fruit were taken from normal ambient storage at 0 °C before starting the experiment.	Validation at 0 °C (see Fig. 1)
D3	15/10/2010	Fruit were taken from normal ambient storage at -1 °C before starting the experiment.	Validation at 20 °C (see Fig. 1). Data was published in Ho et al., (2015).
D4	28/10/2010	Fruit were taken from normal ambient storage at -1 °C before starting the experiment.	Validation at 10 °C. Data was published in Ho et al., (2015).
D5	16/11/2016	Fruit were taken from normal ambient storage at 0 °C before starting the experiment.	Validation at 20 °C (see Fig. S8).
D6	07/12/2016	Fruit were taken from normal ambient storage at 0 °C before starting the experiment.	Validation at 10 °C (see Fig. S8).
D7	16/11/2016	Fruit were taken from normal ambient storage at 0 °C before starting the experiment.	Validation at 5 °C (see Fig. S8).

$$R + mO_2 \underset{k_{H2}}{\overset{k_{H1}}{\rightleftharpoons}} R_a \quad \text{R1}$$

$$R_a + [\mathrm{AA}] \xrightarrow{k_s} E + R_a \quad \text{R2}$$

$$E \xrightarrow{k_d} I \quad \text{R3}$$

$$R_a \xrightarrow{k_{d2}} R_I \quad \text{R4}$$

$$\frac{\partial [R_a]}{\partial t} = k_{H1} \cdot [R] \cdot [O_2]^m - (k_{H2} + k_{d2}) \cdot [R_a] \quad \text{Eq1}$$

$$\frac{\partial [E]}{\partial t} = k_s \cdot [R_a] \cdot [\mathrm{AA}] - k_d \cdot [E] \quad \text{Eq2}$$

Fig. S1. Proposed reactions and modelled equations describing response of receptor, enzyme and respiration to O_2 level. In the signal-enzyme mechanism (reaction R1), the receptor *R* is assumed to be activated or inhibited by O_2 level and described by Hill equation while the concentration of the enzyme *E* is controlled by the synthesis rate k_s agglomerating multiple conversion steps including transcription and translation. Eq1 and Eq2 in Fig. S1 represent time dependent concentrations of the receptor and enzyme in response to O_2 level. Symbols are defined in Supplemenary Text S1.

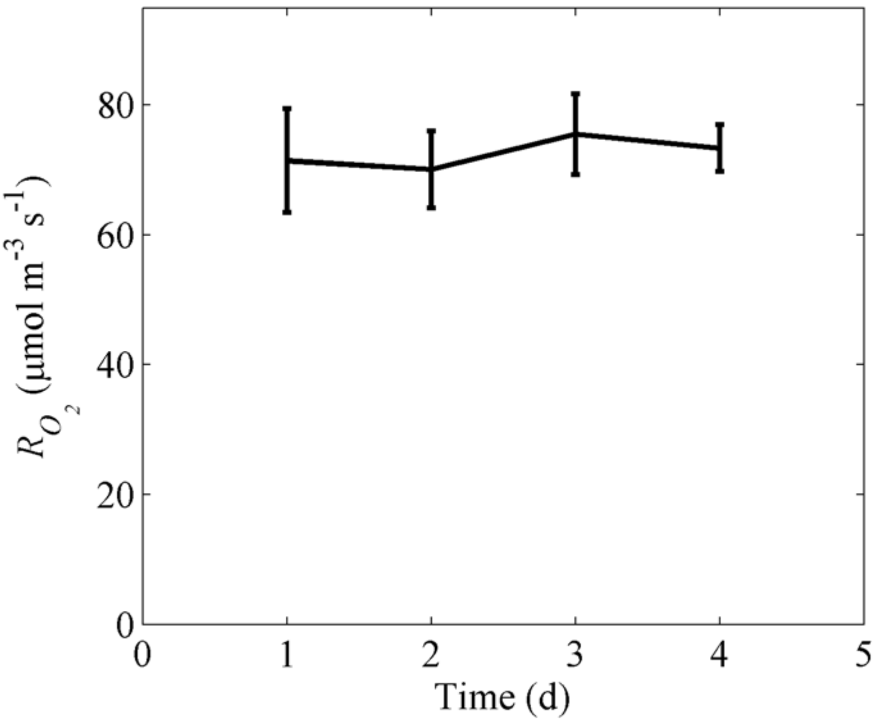

Fig. S2. O_2 consumption rate of intact pear fruit as a function of time at 20 kPa O_2, 0 kPa CO_2 at 10 °C.

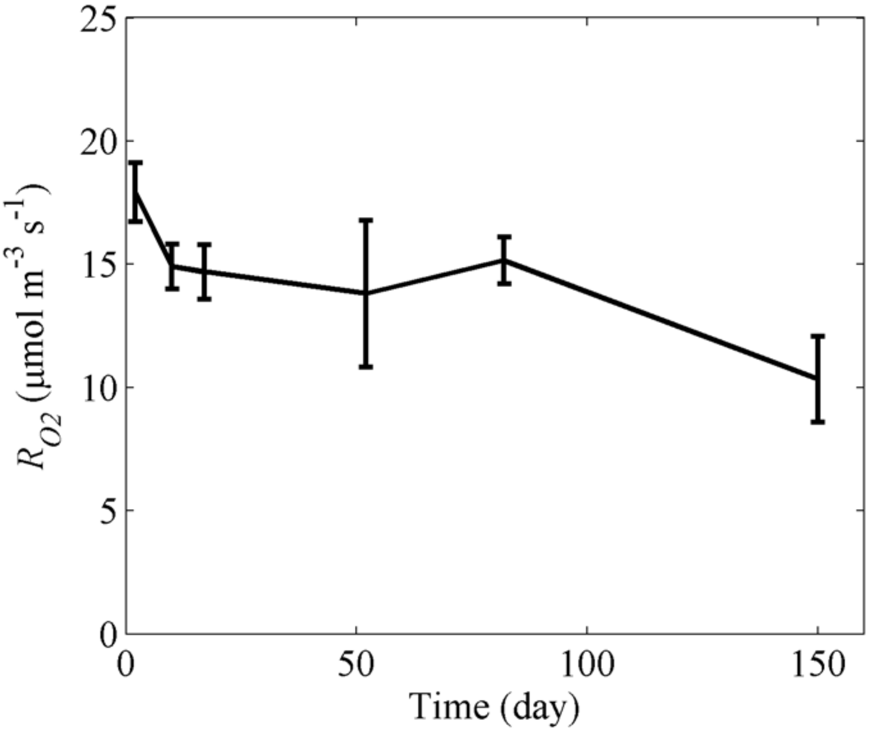

Fig. S3. O_2 consumption rate of intact pear fruit as a function of time during storage of fruit at 20 kPa O_2, 0 kPa CO_2 at 0 °C.

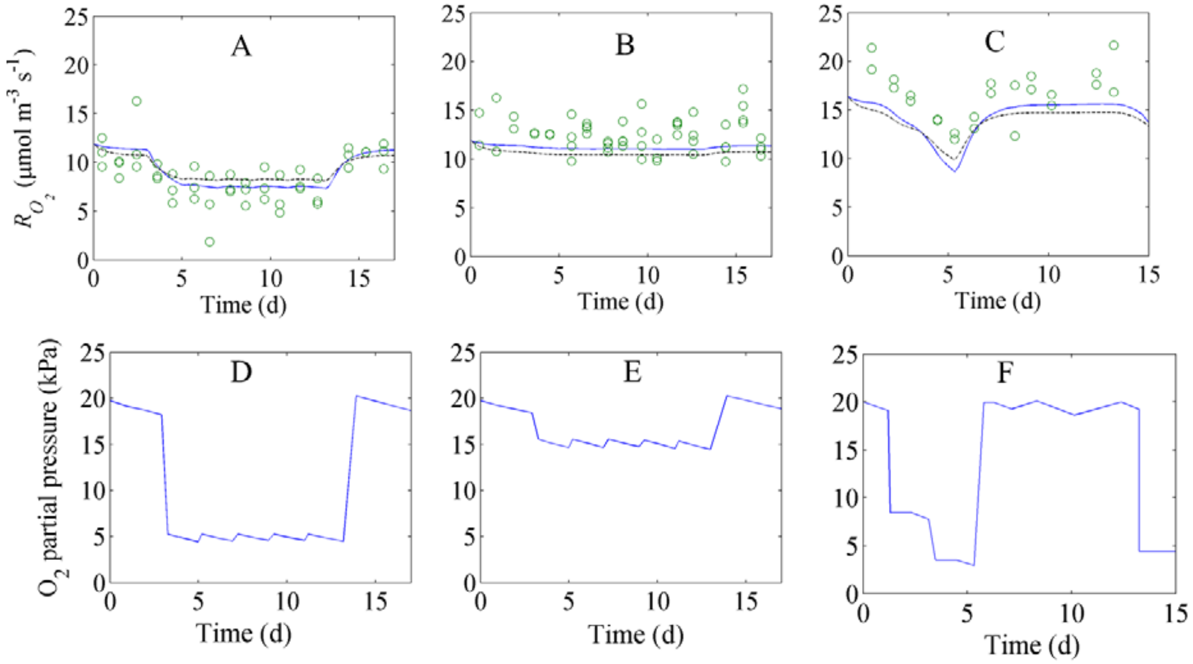

Fig. S4. Dynamic response of O₂ consumption rate (R_{O_2}) to O₂ level and time at 0 °C (experiment B). Panels (A, B, C) and (D, E, F) are R_{O_2} and external O₂ level as a function of time, respectively. Symbols (o) indicate measurements while dashed (– –) and solid (—) lines show model predictions with *m* equal to 1 and 2, respectively.

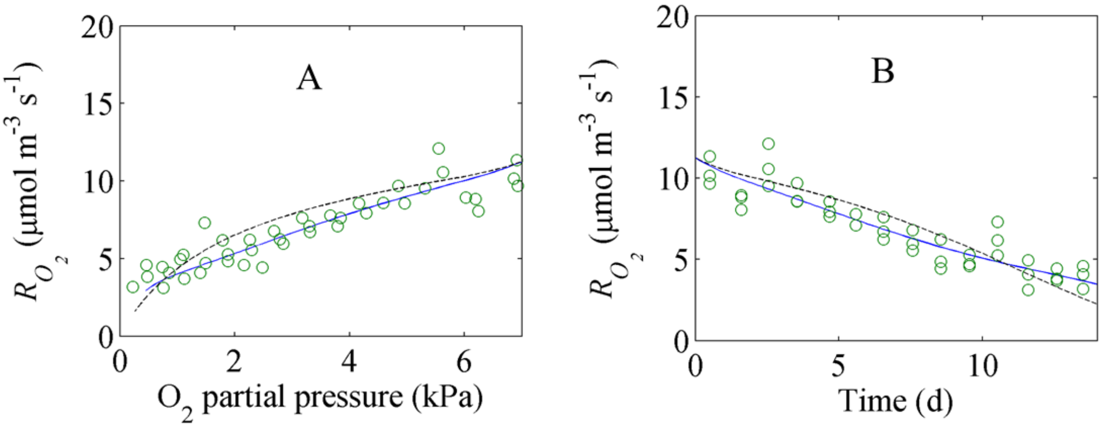

Fig. S5. Response of R_{O_2} of intact pear fruit to O_2 (A) and time (B) (experiment C). Symbols (o) indicate measurements while dashed (– –) and solid (—) lines show model predictions with *m* equal to 1 and 2, respectively.

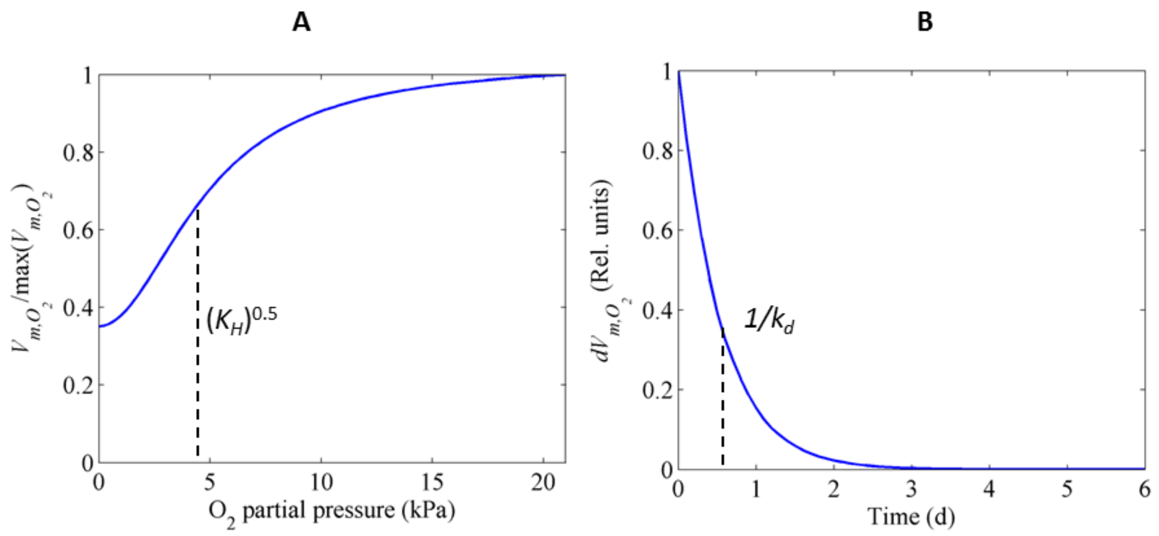

Fig. S6. (A) Steady state modelled response of relative maximal O2 consumption rate ($V_{m,O_2}/\max(V_{m,O_2})$ **) to O2 level.** At a steady state $V_{m,O_2} = V_R$. **(B) Change of maximal O2 consumption rate in response to a sudden drop of the O2 concentration.** $dV_{m,O_2} = \dfrac{V_{m,O_2} - V_R}{V_{m,O_2,ini} - V_R}$, where V_{m,O_2} and $V_{m,O_2,ini}$ are the maximal O2 consumption rate at time t and initial time; V_R is the maximal O2 consumption rate at a steady O2 concentration level (defined in Eq S13). k_d and K_H are defined in Eqs S6 and S9, respectively.

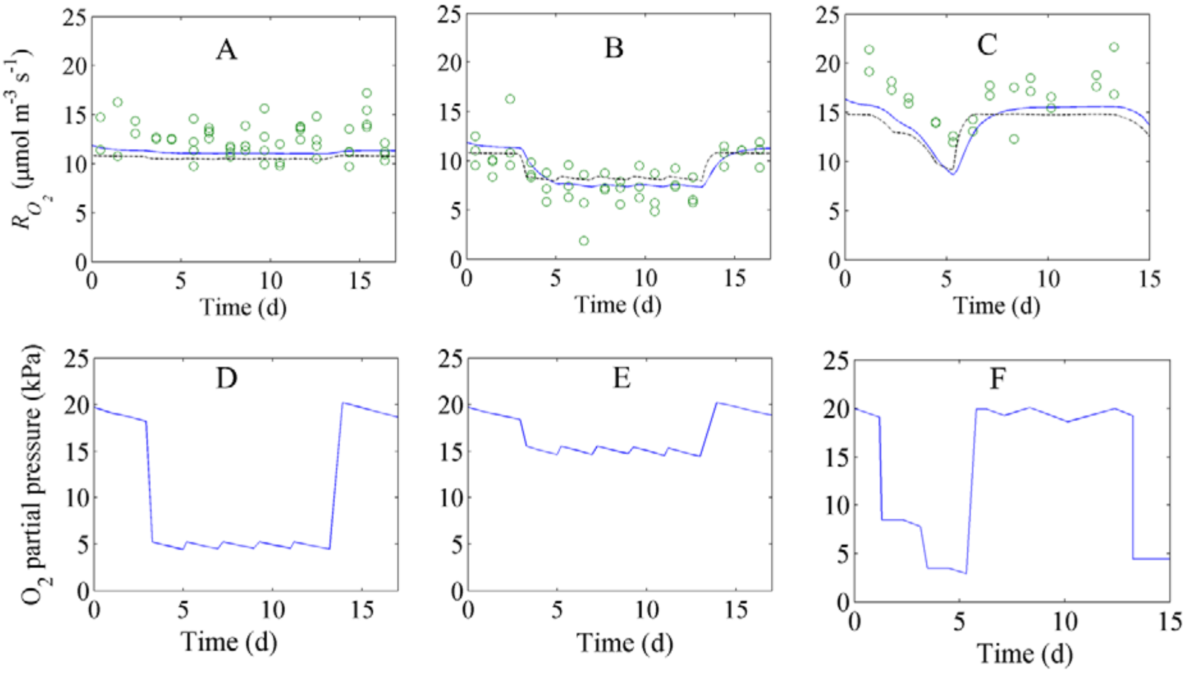

Fig. S7. Comparison of fitting between the adapted respiration model (*m* = 2) and the respiration model with assumption of variation of K_{m,O_2} at 0 °C. Panels (A, B, C) and (D, E, F) are R_{O_2} and external O2 level as a function of time, respectively. Symbols (o) indicate measurements (experiment B) while dashed (– –) and solid (—) lines show model with variation of K_{m,O_2} and the adapted respiration model, respectively.

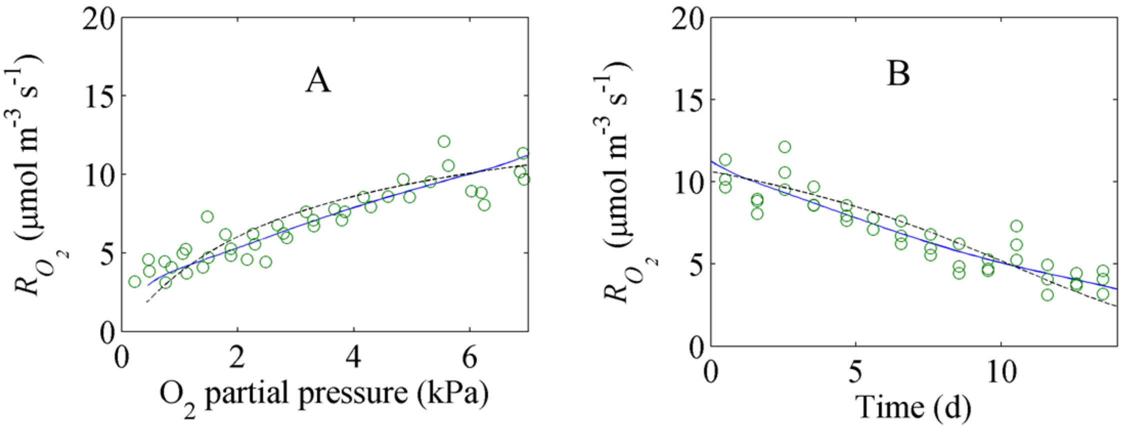

Fig. S8. Comparison of fitting between the adapted respiration model (*m* = 2) and the respiration model with assumption of variation of K_{m,O_2} at 0 °C. Symbols (o) indicate measurements (experiment C) while dashed (– –) and solid (—) lines show model with variation of K_{m,O_2} and the adapted respiration model, respectively.

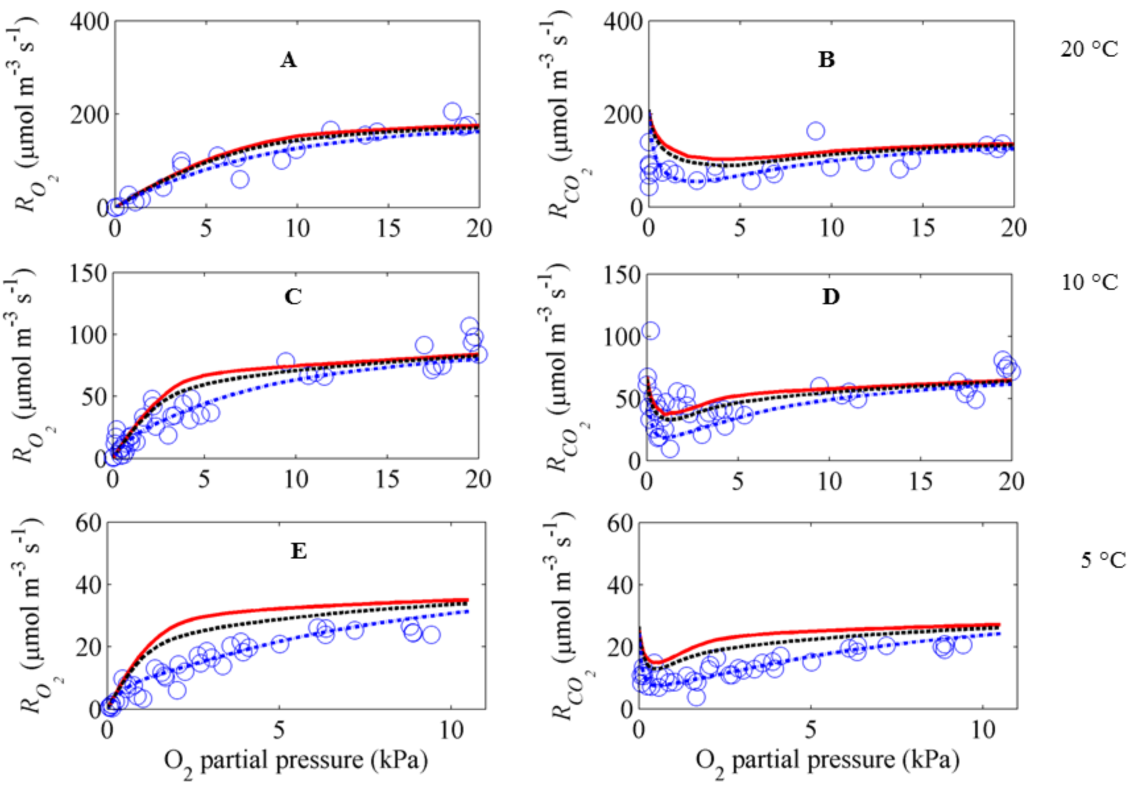

Fig. S9. Respiration rate of intact pear fruit as a function of the O_2 concentration at 20 °C (A, B), 10 °C (C, D), and 5 °C (E, F) harvested in season 2016. R_{O_2} and R_{CO_2} are the O_2 consumption rate and CO_2 production rate, respectively. Symbols (o) indicate measurements (experiment D). Solid red lines (—), dashed black lines (− −) and dotted blue lines (···) correspond to simulations with assumed $\Delta V / V_{R,2}$ of 0, 0.21 and 0.66, respectively. Ratio $\Delta V / V_{R,2}$ represents the amplitude of regulation of maximal respiration rate by O_2 (see in Supplementary text S1 for its derivation). Measurements were carried out in the season of 2016.

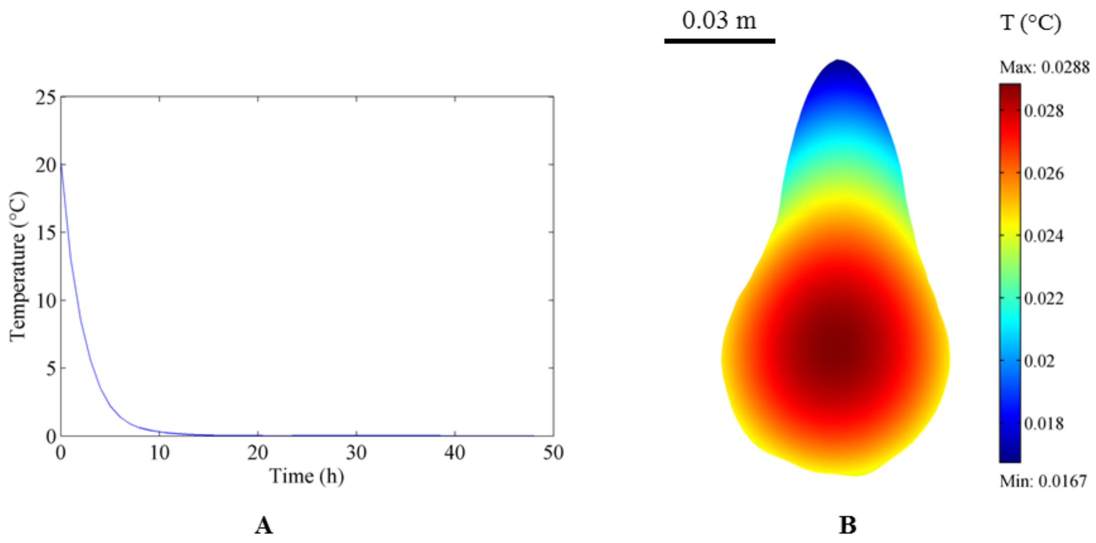

Fig. S10. Predicted temperature of pear fruit during cooling. (**A**) Mean fruit temperature as a function of time during cooling from 20 °C to 0 °C; (**B**) Temperature profile within the pear at steady state at 0 °C. The maximal temperature difference is about 0.01 °C. The temperature is not completely equal to 0 °C because of the heat production caused by respiration.

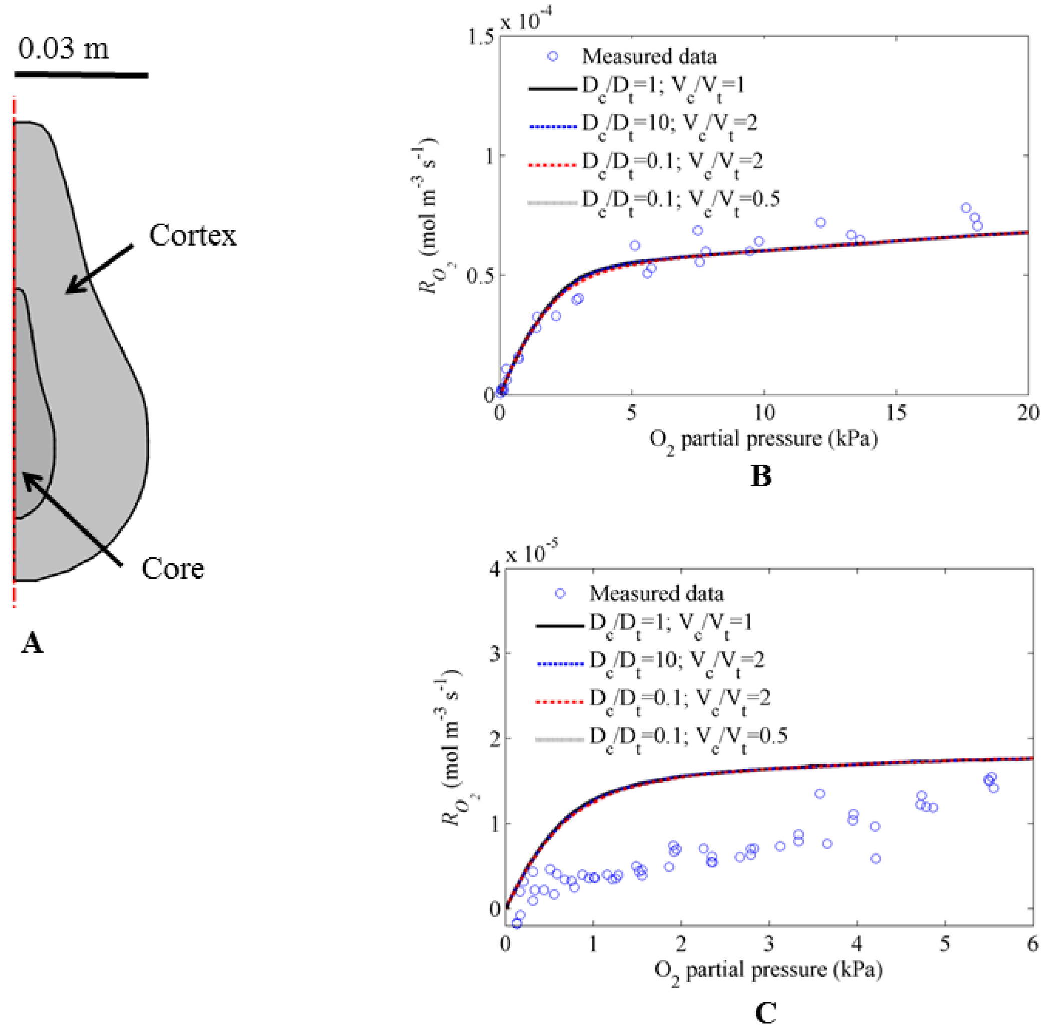

Fig. S11. Simulations with a two-compartment model (core and cortex) and different combinations of diffusivities and V_{max} values. (A) 2D axisymmetric model of the pear fruit. The core was obtained by scaling the fruit to 30% of its origin size. The volume of the core occupied 4.5% of the fruit volume. (B) and (C) show the O_2 consumption rates of intact pear fruit as a function of O_2 concentrations at 10 °C and 0 °C, respectively. Symbols indicate the measured data while lines represent simulations. D_c/D_t is the ratio of the diffusivity of the core (D_c) to that the cortex (D_t) while V_c/V_t is the ratio of the maximal respiration rate of the core (V_c) to that the cortex (V_t). Values of D_t and V_t were taken from Table 1 and Table 2, respectively while values of D_c and V_c were assumed to vary. Respiration was assumed to follow Michaelis Menten kinetics

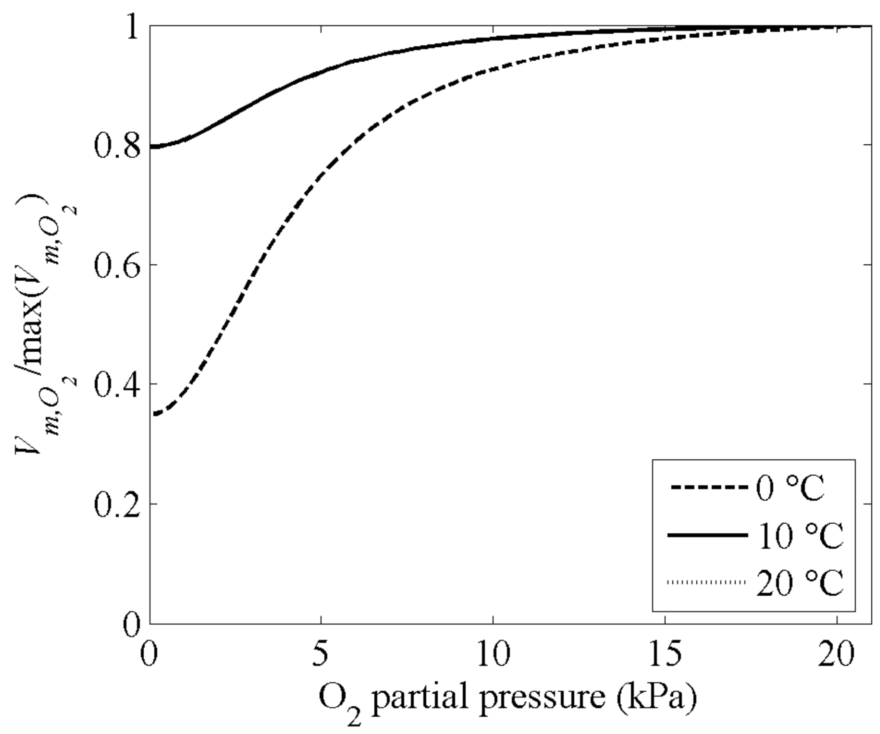

Fig. S12. Steady state modelled response of relative maximal O2 consumption rate (V_{m,O_2} $/\max(V_{m,O_2})$) to O2 level. ΔV which represents response of the maximal respiration rate to O2 level was assumed to be equal to $0.66 \cdot V_{R,2}$, $0.21 \cdot V_{R,2}$ and $0.21 \cdot V_{R,2}$ at 0 °C, 10 °C and 20 °C, respectively. The curve at 10 °C is coincident to that at 20 °C.

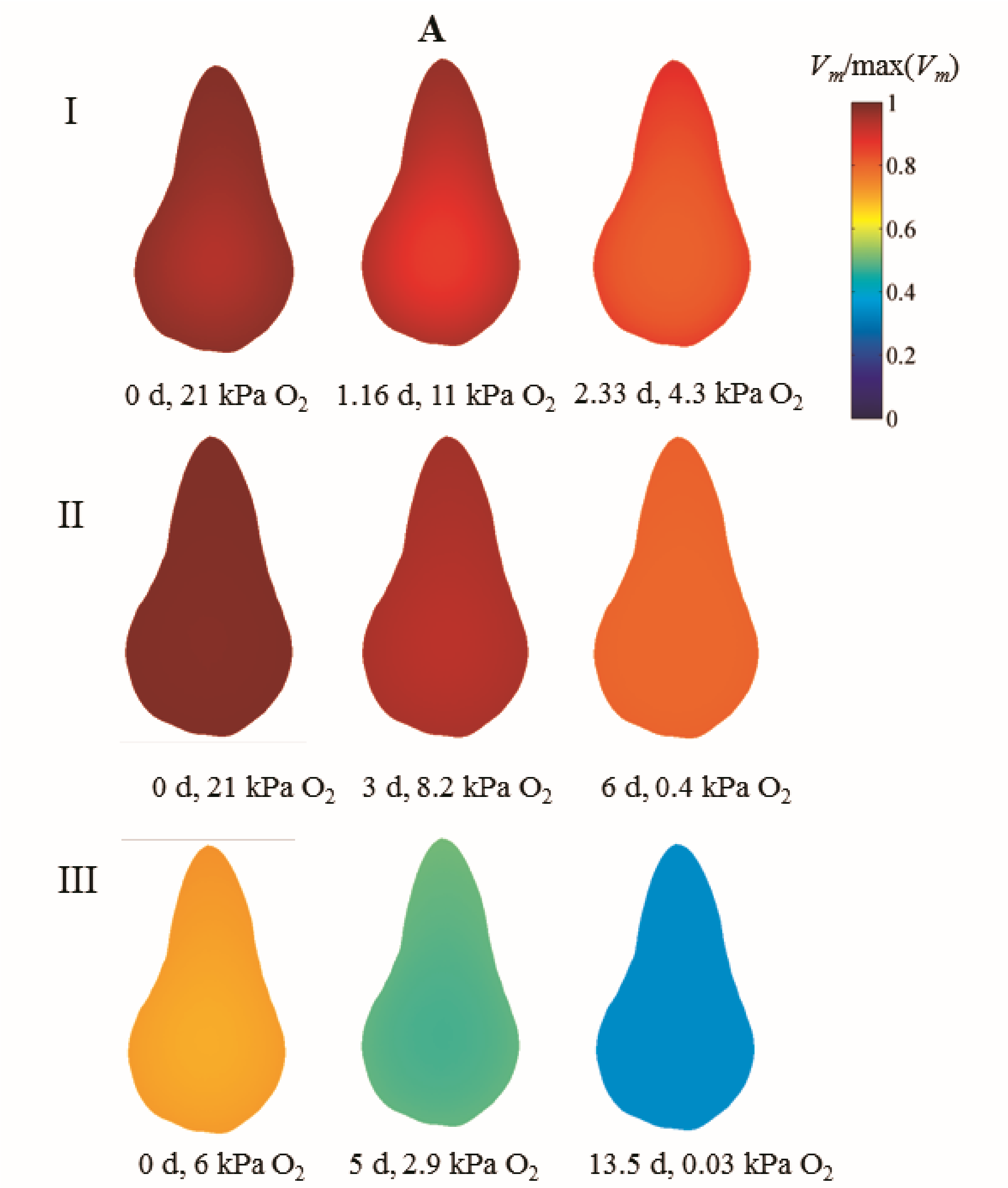

Fig. S13. Simulated V_{m,O_2} of pear fruit in the closed jar at 20 °C (I), 10 °C (II) and 0 °C (III) and different times. The color map represents $V_{m,O_2} / \max(V_{m,O_2})$. In the simulations, Ratio $\Delta V / V_{R,2}$ representing amplitude of regulation of maximal respiration rate by O_2 (see in Supplementary text S1 for its derivation) was assumed to be 0.21, 0.21 and 0.66 at 20 °C, 10 °C and 0 °C, respectively. Simulated V_{m,O_2} corresponds to O_2 and CO_2 partial pressures of pear fruit described in Fig. 2.

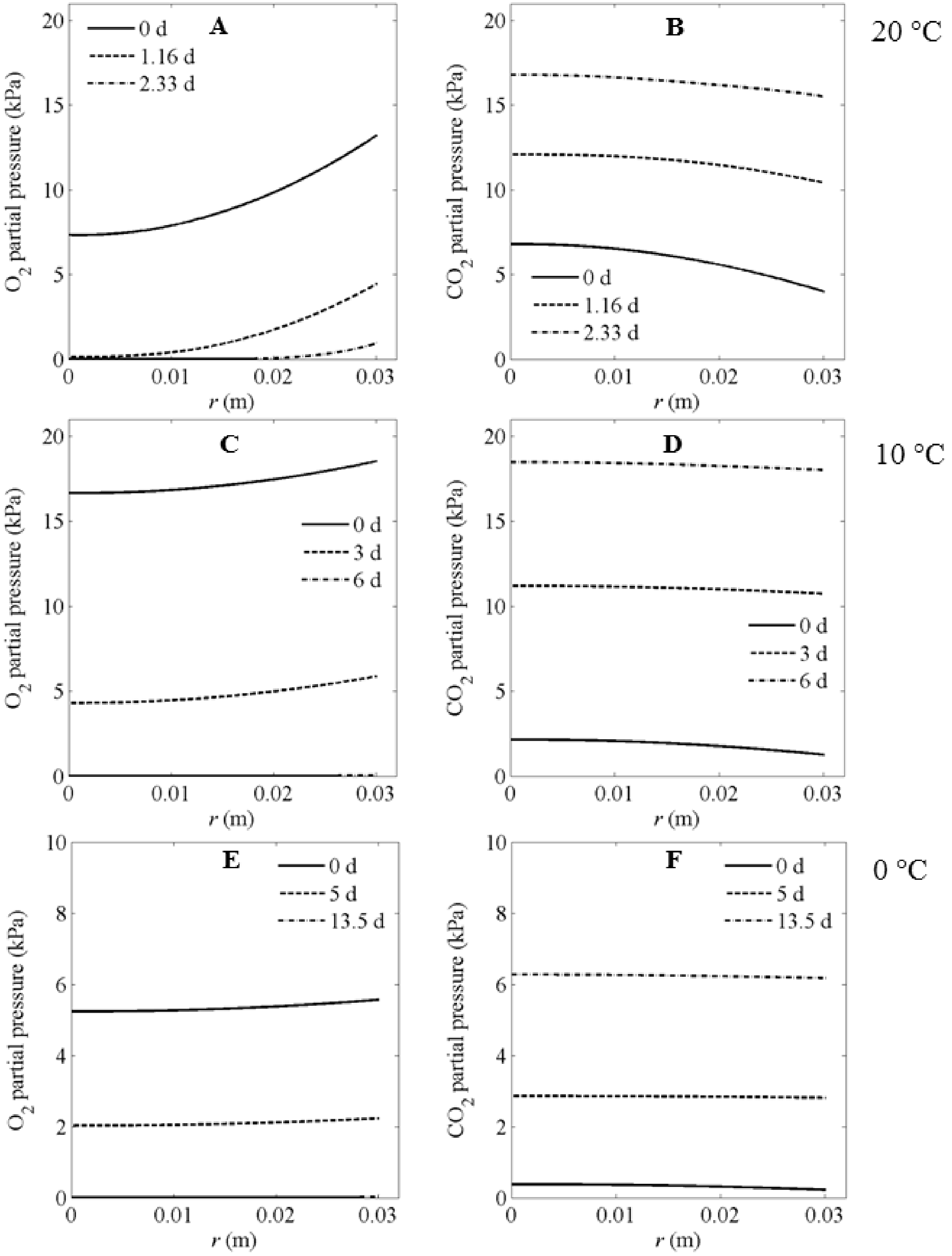

Fig. S14. Simulated O_2 and CO_2 gas partial pressure profiles from the center to the surface along the radial direction in the closed jar at 20 °C (I), 10 °C (II) and 0 °C (III) at different times (where responses of respiration rate to time and O_2 level were described in Fig.3).

References:

Hill AV. 1910. The possible effects of the aggregation of the molecules of hæmoglobin on its dissociation curves. The Journal of Physiology **40**, 01-22 (Suppl): iv–vii. *doi*:*10.1113/jphysiol.1910.sp001386.*

Ramaswamy HS, Tung MA. 1981. Thermophysical properties of apples in relation to freezing. Journal of Food Science **46**, 724–728.

Rao MA, Rizvi, SSH, Datta AK. 2005. *Engineering Properties Of Foods*. CRC Press, New York.
